# LncRNA TIALD contributes to hepatocellular carcinoma metastasis via inducing AURKA lysosomal degradation

**DOI:** 10.1038/s41420-023-01620-w

**Published:** 2023-08-26

**Authors:** Yingchao Wang, Yue Zhong, Xiaoyuan Zheng, Niangmei Cheng, Yong Yang, Ye Yang, Fei Wang, Qiuyu Zhuang, Yao Huang, Wuhua Guo, Naishun Liao, Xiaoyu Yang, Bixing Zhao, Xiaolong Liu

**Affiliations:** 1 The United Innovation of Mengchao Hepatobiliary Technology Key Laboratory of Fujian Province, Mengchao Hepatobiliary Hospital of Fujian Medical University, Fuzhou, 350025 P. R. China; 2 Fujian Provincial Clinical Research Center for Hepatobiliary and Pancreatic Tumors, Fuzhou, 350025 P. R. China; 3 Mengchao Med-X Center, Fuzhou University, Fuzhou, 350116 P. R. China; 4 College of Life Science, Fujian Agriculture and Forestry University, Fuzhou, 350002 P. R. China; 5 Fuzhou Hospital of Traditional Chinese Medicine Affiliated to Fujian University of Traditional Chinese Medicine, Fuzhou, 350001 China

**Keywords:** Prognostic markers, Metastasis, RNA

## Abstract

The N6-methyladenosine (m6A) RNA methyltransferase METTL16 is an emerging player in RNA modification landscape and responsible for the deposition of m6A in a few transcripts. AURKA (aurora kinase A) has been confirmed as an oncogene in cancer development including hepatocellular carcinoma (HCC). Nevertheless, it remains unclear whether METTL16 mediated m6A modification of lncRNAs can regulate AURKA activation in cancer progression. Here we aimed to investigate the functional links between lncRNAs and the m6A modification in AURKA signaling and HCC progression. Here we show that LncRNA TIALD (transcript that induced AURKA Lysosomal degradation) was down-regulated in HCC tissues by METTL16 mediated m6A methylation to facilitate its RNA degradation, and correlates with poor prognosis. Functional assays reveal that TIALD inhibits HCC metastasis both in vitro and in vivo. Mechanistically, TIALD directly interacts with AURKA and facilitate its degradation through the lysosomal pathway to inhibited EMT and metastasis of HCC. AURKA’s specific inhibitor alisertib exerts effective therapeutic effect on liver cancer with low TIALD expression, which might provide a new insight into HCC therapy. Our study uncovers a negative functional loop of METTL16-TIALD-AURKA axis, and identifies a new mechanism for METTL16 mediated m6A-induced decay of TIALD on AURKA signaling in HCC progression, which may provide potential prognostic and therapeutic targets for HCC.

## Introduction

N6-methyladenosine (m6A) is the most common and abundant epigenetic modification in eukaryotic mRNAs and non-coding RNAs [1]. It has been demonstrated that m6A modification played critical roles in regulating RNA stability, location and translation at post transcriptional level [2, 3]. METTL16 is a newly discovered and limiting understanded RNA methyltransferase, and it was only been proven to catalyze m6A installation in a few non-coding RNAs: U6 small nuclear RNA, MALAT1 and XIST, and mRNA target (MAT2A) [4–6]. It still requires more strongly evidences to prove that METTL16 participates in the regulation of cancer progression through inducing m6A modification of mRNAs or ncRNAs.

Long non-coding RNAs (lncRNAs) are transcripts consisting of nucleotides >200 with lacking of protein coding potential [7], which were ever deemed as transcriptional noise. However, to date, increasing evidence indicates that lncRNAs are implicated in various biological processes including cell proliferation, differentiation and metastasis [8]. Aberrant expression of lncRNAs has frequently been identified as biomarkers, prognostic predicators in multiple cancer types [9, 10]. Furthermore, many lncRNAs have been demonstrated to play versatile roles in both facilitating or suppressing tumorigenesis and cancer progression [11]. Recently, more and more studies have revealed that lncRNA m6A modifications were associated with tumorigenesis, metastasis, and other tumor characteristics. However, the role of lncRNA m6A modification in hepatocellular carcinoma (HCC) progression remains limited understanding.

AURKA (Aurora kinase A) belongs to the family of serine/threonine-protein kinase and is essential for cell cycle progression. AURKA shows significantly higher expression in cancer tissues than in normal tissues for multiple tumor types including HCC [12]. AURKA functions as an oncogene by regulating multiple molecular targets that involved in the regulation of cancer proliferation, apoptosis, metastasis, genomic instability and stemness [13]. AURKA overexpression is likely to be regulated not only by gene amplification, but also by other mechanisms such as transcriptional activation and suppression of protein degradation [14]. A large number of proteins have been reported to be involved in regulating the degradation of AURKA. For example, AURKA protein stability is maintained by Twist [15], ALDH1A1 [16], YBX1 [17] and the deubiquitinase USP2a [18] through ubiquitin-proteosomal degradation pathway. SMAD4 induces the ubiquitin proteasomal degradation of AURKA [19]. Whether LncRNA and its m6A modifications are involved in regulating AURKA activation still remains poorly understood.

In the present study, we show that a specific lncRNA AC079360.1, whose expression is significantly decreased in HCC tissues comparing to that in non-tumor tissues, is significantly correlated with poor prognosis of HCC patients. Functional assays demonstrate that the AC079360.1 plays important roles in inhibiting metastasis of HCC cells by inducing AURKA lysosomal degradation. For convenience, we refer to AC079360.1 as transcript that induced AURKA Lysosomal degradation (TIALD) in this study. Mechanistically, METTL16-mediated m6A modification reduces the stability of TIALD resulting in loss of biological function of TIALD. More importantly, alisertib, a specific AURKA inhibitor, could well inhibit the metastasis of HCC cells with low TIALD expression in vitro and in vivo. Taken together, we identified a novel m6A-mediated lncRNA, TIALD, which is the first lncRNA shown to be involved in the regulation of AURKA lysosomal degradation, links the function of METTL16 induced m6A modification on promoting HCC metastasis. Our study provided a solid theoretical basis for the clinical translation of antitumor drugs targeting AURKA and extend the understanding of the importance of METTL 16 mediated m6A RNA modification in cancer biology.

## Results

### LncRNA TIALD is down-regulated in HCC tissues and associated with poor prognosis

**Fig. 1:**
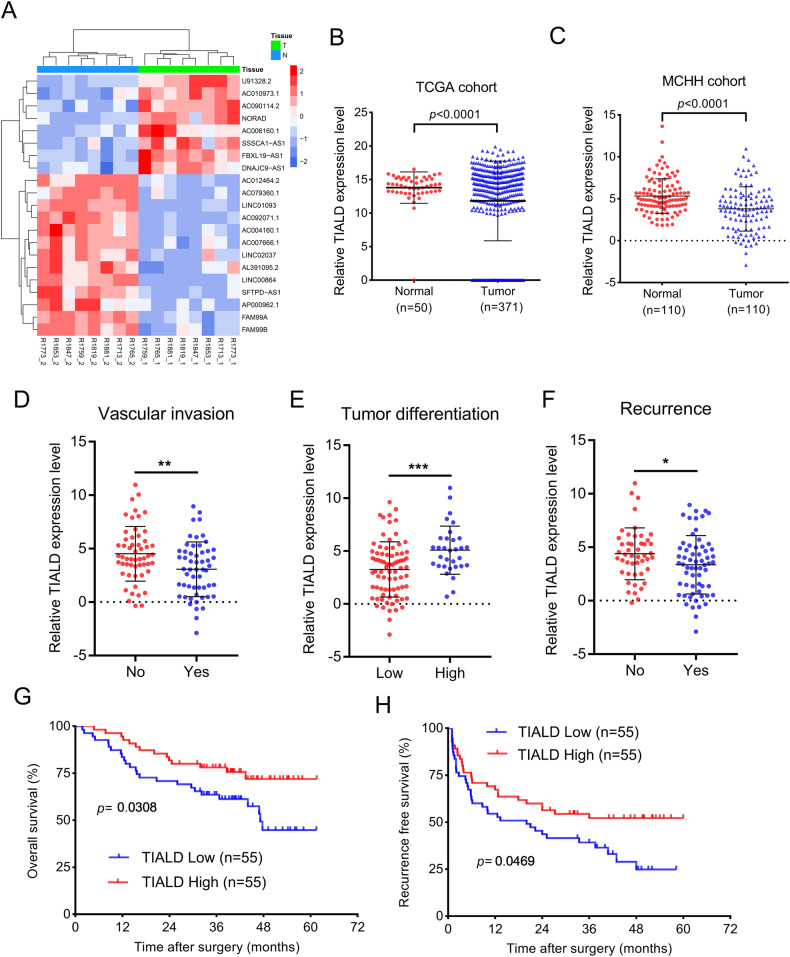
TIALD expression is down-regulated and associated with prognosis in HCC. **A** Heat-map of the 21 differentially expressed lncRNAs in 8 paired HCC tumor and adjacent para-tumor tissues. **B** Relative expression level of TIALD in adjacent para-tumor tissues (*n* = 50) and tumor tissues (*n* = 371) from TCGA cohort. **C** RT-qPCR validation of TIALD expression in another cohort of 110 HCC patients with paired tumor and para-tumor tissues. 18 S rRNA was used as internal control. Student’s *t*-test was used to compare the difference between para-tumor and tumor tissues. **D** Relative expression of TIALD in HCC patient with or without vascular invasion. **E** Differential expression levels of TIALD in HCC patient with low differentiation and high differentiation. **F** Relative expression of TIALD in HCC patient with recurrence or not. **G**, **H** Kaplan–Meier analysis revealed that low expression of TIALD significantly associated with shorter Overall survival (**G**) and Recurrence free survival (**H**).

To identify lncRNAs involved in the tumorigenesis and progression of HCC, a transcriptome analysis was firstly performed in 8 pairs of HCC and their adjacent non-tumor tissues. A total of 21 differentially expressed lncRNAs were identified with 8 upregulation and 13 downregulation (Fig. 1A). Among these lncRNAs, the AC079360.1 (TIALD) was identified to be down-regulated in HCC tissues compared with that in their paired adjacent non-tumor tissues. The downregulation of TIALD was also observed in the TCGA dataset (Fig. 1B). Subsequently, the expression of TIALD was further investigated in an enlarged sample cohort including 110 pairs of HCC and their adjacent non-tumor tissues. The results showed that TIALD was significantly down-regulated in HCC tissues compared with that in non-tumor tissues (Fig. 1C). Thereafter, the samples used for qRT-PCR assay were divided into high group and low group depending on the median value of TIALD expression in HCC tissues. Further clinical pathology data analysis shows that the decreased expression of TIALD significantly correlated with vascular invasion, differentiation and recurrence (Table 1, Fig. 1D-F). More importantly, Kaplan–Meier analysis demonstrated that the decreased expression of TIALD was significantly associated with shorter overall survival (OS, *p* = 0.0308, Fig. 1H) and recurrence free survival (RFS, *p* = 0.0469, Fig. 1G). Additionally, the Cox regression model indicated that TIALD as an independent risk factor for predicting OS and RFS of HCC patients (Table S4). Collectively, these results indicated that TIALD might be implicated in HCC metastasis or recurrence and serves as a prognostic predictor for HCC patients.

**Table 1:** Association between TIALD expression and clinicopathological features of patients with HCC (*n* = 110).

Parameter		No. of patients	TIALD expression		*χ*^*2*^*-*value	*P*-value
			Low(*n* = 55)	High(*n* = 55)		
Sex	male	90	44	46	0.244	0.621
	female	20	11	9		
Age (year)	<55	59	32	27	0.914	0.339
	≥55	51	23	28		
Tumor size (cm)	<5	58	27	31	0.584	0.445
	≥5	52	28	24		
Vascular invasion	Yes	55	33	22	**4.400**	**0.036**
	No	55	22	33		
Tumor number	Single	107	55	52	3.084	0.079
	Multiple	3	0	3		
AFP (ng/ml)	≤400	77	36	41	1.082	0.298
	>400	33	19	14		
Differentiation grade	I–II	32	11	21	**4.407**	**0.036**
	III–IV	78	44	34		
TNM stage	I–II	85	41	44	0.466	0.495
	III–IV	25	14	11		
Liver cirrhosis	yes	88	47	41	2.045	0.153
	no	22	8	14		
Recurrence	Yes	63	37	26	**4.495**	**0.034**
	No	47	18	29		

*TNM stage* Tumor-Node-Metastasis stage, *AFP* alpha-fetoprotein.

Chi-square test was used to compare the statistical differences between the high expression and low expression of AC079360.1. *P* < 0.05 was considered as statistically significant.

### TIALD inhibits HCC metastasis both in vitro and in vivo

**Fig. 2:**
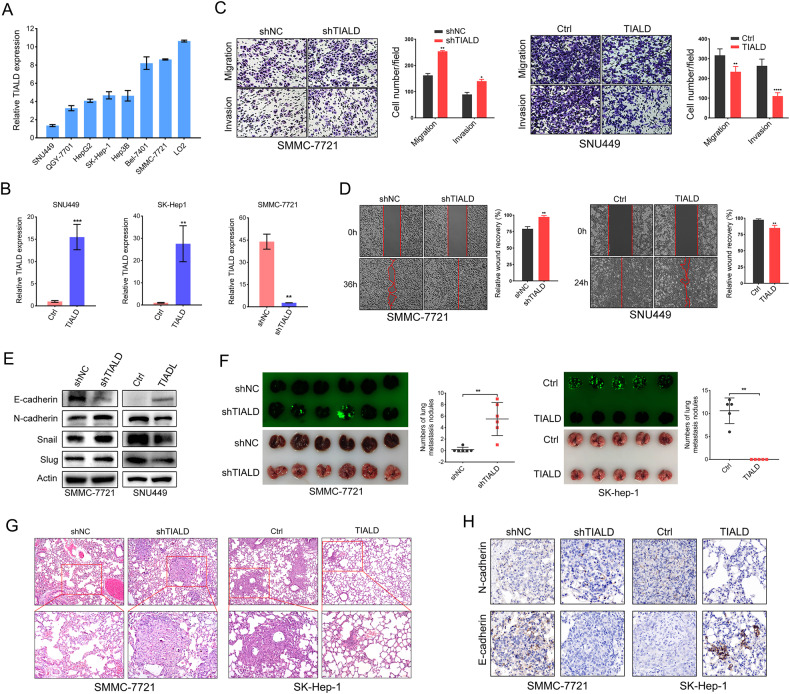
TIALD inhibits HCC metastasis both in vitro and in vivo. **A** Relative expression of TIALD in LO2 and different liver cancer cell lines. **B** Expressions of TIALD in SNU449 and SK-Hep-1 cells stably transfected with TIALD or Control and in SMMC-7721 cells stably transfected with shTIALD or shNC were detected by real-time qRT-PCR. **C** Representative and quantification results of transwell cell migration and invasion assay in SMMC-7721 and SNU449 cells. **D** Representative and quantification results of wound healing assay. **E** Western blotting detection of epithelial (E-cadherin), mesenchymal (N-cadherin) and transcription factors (Slug and Snail) in SMMC-7721 and SNU449 cells. **F** Representative images and quantification data of metastatic nodules in the lung tissues of mice. Stable cell lines of TIALD over-expression or knockdown were injected by tail vein in B-NDG mice. **G** Representative images of lung metastatic nodules stained with H&E. **H** Representative images of IHC staining in lung metastasis nodules of mice.

Next, we examined the expressions of TIALD in seven human hepatoma cell lines and a normal liver cell line, LO2. The results showed that the expression of TIALD in LO2 cells was the highest in all cell lines, among which SMMC-7721 and SK-Hep-1 exhibited a relatively higher and medium TIALD expression, whereas SNU-449 displayed considerably lower TIALD expression (Fig. 2A).

To explore the potentially biological functions of TIALD in regulating the mobility and invasiveness of HCC cells, TIALD was artificially over-expressed in SNU449 and SK-Hep-1 cells and knocked down in SMMC-7721 cells respectively. And successful construction of stably modified cell lines was confirmed by qRT-PCR assay (Fig. 2B). Notably, TIALD over-expression in SNU449 cells dramatically inhibited the cell migration and invasion in vitro (Fig. 2C). Correspondingly, knockdown of TIALD in SMMC-7721 cells dramatically increased the abilities of migration and invasion (Fig. 2C). Wound-healing assays further confirm that TIALD plays an important role in suppressing the migratory abilities of HCC cells (Fig. 2D). Additionally, cell cycle and cell apoptosis assay were also performed and there was not significant difference in both TIALD overexpressed cells and TIALD knock-down cells (Fig. S1).

EMT is well known to be involved in the invasion and metastasis of cancer cells [20], we next investigated whether TIALD is involved in the regulation of EMT in HCC. Western blotting showed that knockdown of TIALD in SMMC-7721 cells dramatically promoted the expression of mesenchymal markers N-cadherin, Slug and Snail, but decreased the expression of epithelial marker E-cadherin (Fig. 2E). Consistently, opposite results were obtained in SNU-449 with TIALD over-expression (Fig. 2E). Therefore, our results indicated that TIALD inhibited the migration and invasion of HCC cells through inactivating EMT.

To further confirm our findings, an in vivo study was performed. Cells with TIALD over-expression or knockdown were injected into mice tail vein to construct lung metastasis model. The results showed that the number of lung metastatic foci in TIALD knockdown group were remarkably more than that in control group as shown by fluorescent spots (Fig. 2F). Hematoxylin and eosin (H&E) staining confirmed that control group had significantly less metastatic nodules than TIALD knockdown group (Fig. 2G). Furthermore, the size of the nodules in control group was remarkably smaller than those in TIALD knockdown group. Conversely, metastatic nodules were hardly observed in TIALD over-expression group unlike in control group (Fig. 2F and G). In the mouse lung metastases nodules, we performed the immunohistochemical staining, as shown in Fig. 2H, the expression of E-cadherin is relatively higher in metastatic nodules with TIALD over-expression but lower in TIALD knockdown group. Conversely, the expression of N-cadherin showing the opposite trend (Fig. 2H).

Collectively, these results indicate that TIALD is capable of regulating EMT and metastasis phenotype of HCC both in vitro and in vivo.

### METTL16 mediated downregulation of TIALD in HCC

**Fig. 3:**
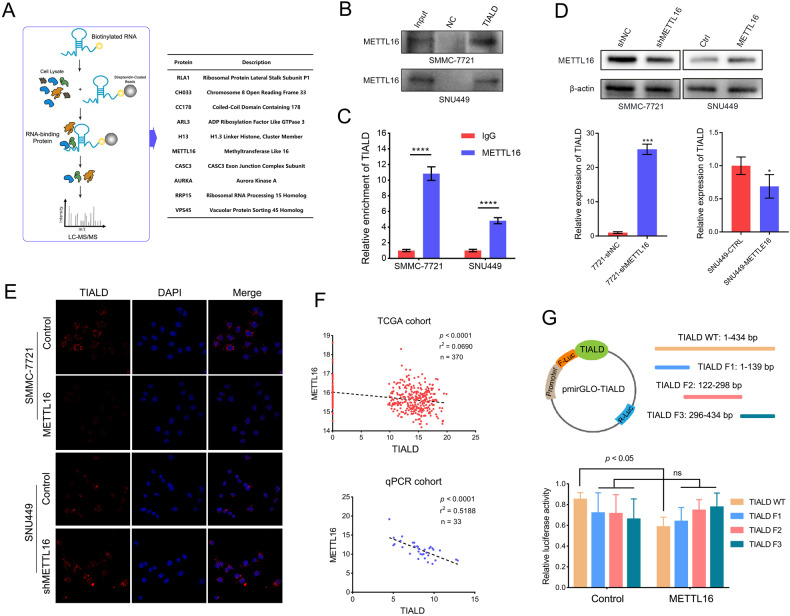
METTL16 mediated downregulation of TIALD in HCC. **A** LncRNA pulldown combined with mass spectrometry to Identify TIALD interaction proteins (left) and list of potential interacting proteins of TIALD (right). **B** RNA pull-down assay was performed in SMMC-7721 and SNU449 cells using biotin-labeled TIALD RNA probe transcribed in vitro, and METTL16 was detected using western blots. **C** RIP-qPCR analysis of the interaction of METTL16 and TIALD in SMMC-7721 and SUN449 cells. Enrichment of TIALD with METTL16 antibody and IgG control was measured by qPCR. **D** METTL16 was knock-down or over-expressed in SMMC-7721 and SNU449 cells, the expression of METTL16 was detected by western blots and the expression of TIALD was detected by qPCR. **E** FISH analysis of the expression of TIALD in METTL16 over-expression SMMC-7721 cells and METTL16 knockdown SNU449 cells. **F** The correlation between METTL16 and TIALD in TCGA datasets (up) and qRT-PCR analysis of METTL16 and TIALD in 33 HCC tissues (down). **G** Wild-type and different fragment of TIALD sequences were cloned into a luciferase reporter (up). Relative luciferase activity of the wild-type and different fragment of TIALD reporter vectors catalyzed by METTL16 (down).

It has been reported that N6-methyladenosine (m6A) is one of the most common and abundant RNA modifications in eukaryotic mRNAs and lncRNAs, and functionally modulates RNA metabolic processes, including splicing, localization, translation and stability [21]. To explore whether the downregulation of TIALD expression in HCC is related to methylation modification, RNA pull down/MS was performed to identify whether there are interactions between TIALD and proteins which was participated in RNA methylation modification (Fig. 3A). A silver stain of the IP gel was shown in Fig. S2. Fortunately, among the candidates interacted with TIALD, we have identified METTL16, which is a m6A methyltransferase. Therefore, we speculate that METTL16 could regulate the expressions of TIALD by mediating its m6A modification. To verify our hypothesis, we firstly confirmed the interaction between TIALD and METTL16 using RNA-pull down/western blot and RIP/qRT-PCR (Fig. 3B and C). Furthermore, METTL16 knockdown dramatically improved the expression of TIALD (Fig. 3D). While, the expression of TIALD was inhibited by METTL16 over-expression (Fig. 3D). Fluorescence in situ hybridization (FISH) experiments further confirmed that METTL16 over-expression significantly suppressed the fluorescence intensity of TIALD (Fig. 3E), while METTL16 knockdown enhanced the fluorescence intensity of TIALD (Fig. 3E), demonstrating that METTL16 is involved in the downregulation of TIALD. Meanwhile, clinical tissue sample testing and TCGA data suggested that the expression of METTL16 was negatively associated with the expression of TIALD (Fig. 3F). Dual luciferase assay further showed that METTL16 significantly inhibited the activities of luciferase-TIALD (Fig. 3G) in SNU-449 cells, again confirming the interaction between TIALD and METTL16. To further confirm that METTL16 interacts with a specific region of TIALD, we cloned different TIALD fragments into a luciferase reporter vector, dual luciferase assay showed that over-expression of METTL16 inhibited the luciferase activity of the reporter that containing the full length of TIALD, but did not affect the luciferase activity of these fragments (Fig. 3G), suggesting that METTL16 binding to TIALD requires the full length of the lncRNA, but not a fragment.

These data indicate that METTL16 binds to TIALD and plays important roles in regulating the expression of TIALD.

### m6A modification is involved in the degradation of TIALD

**Fig. 4:**
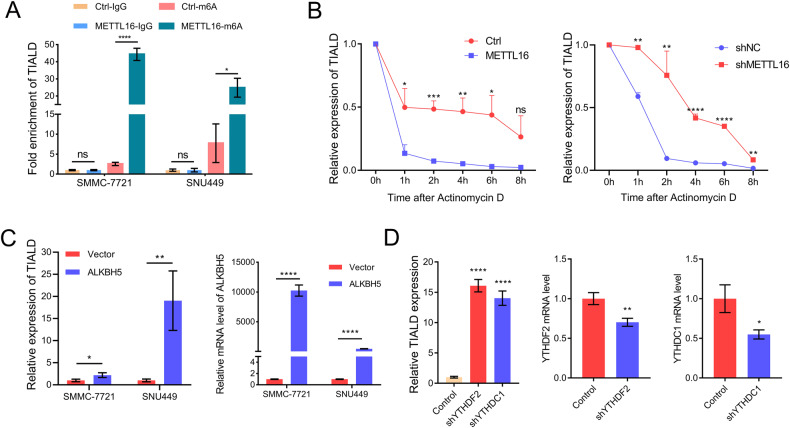
m6A modification is involved in the degradation of TIALD. **A** MeRIP-qPCR analysis was used to demonstrate METTL16-mediated TIALD m6A modification in SMMC-7721 and SNU449 cells. m6A modification of TIALD was significant enriched upon METTL16 over-expression. **B** The expression levels of TIALD in METTL16 or shMETTL16 cells were quantified by real-time PCR at indicated time points after actinomycin D treatment and the decay rate of TIALD was evaluated with a linear regression model. **C** ALKBH5 up-regulated the expression of TIALD. ALKBH5 was over-expressed in SMMC-7721 and SNU449 cells, and the expression of TIALD and ALKBH5 were detected using qPCR. **D** YTHDF2 and YTHDC1 negatively regulated TIALD expression. YTHDF2 and YTHDC1 was knock-down in SMMC-7721 cells and the expressions of TIALD, YTHDF2 and YTHDC1 were detected using qPCR.

Next, we wonder whether m6A modification caused the downregulation of TIALD. me-RIP/qRT-PCR assay showed that METTL16 over-expression significantly increased m6A modification of TIALD (Fig. 4A). Previous studies have shown that m6A methylation regulates RNA metabolism including degradation [22], and we speculate that METTL16-induced TIALD m6A methylation could down-regulate its expression by promoting RNA degradation. To confirm this conjecture, we use actinomycin D to block transcription of cells, our data suggested that knockdown of METTL16 increased the stability of TIALD (Fig. 4B). Supportively, over-expression of METTL16 accelerated the degradation of TIALD (Fig. 4B). Besides m6A writers, the m6A erasers and readers are also involved in m6A remove and recognize the m6A site in RNA. To further identify the m6A erasers and readers that involved in regulating TIALD degradation, ALKBH5, a m6A demethylase [23], was over-expressed in SMMC-7721 and SNU449 cells. As shown in Fig. 4C, ALKBH5 significantly improved the expression of TIALD. Additionally, increasing evidences showed that YTHDF2 and YTHDC1 act as “readers” to accelerate the degradation of m6A-modified RNAs [24, 25]. Therefore, we also examined the expression of TIALD in the cells with knockdown of YTHDF2 or YTHDC1. As our expectation, the expression of TIALD was significantly improved by 16 folds and 14 folds respectively in the cells with knockdown of YTHDF2 or YTHDC1 (Fig. 4D). The RNA decay assay was further performed to confirm that YTHDF2 and YTHDC1 affect the degradation of TIALD (Fig. S3). And the results showed that both knockdown of YTHDF2 and YTHDC1 increased the stability of TIALD.

To further confirm the methylation sites in TIALD, we analyzed the sequence of TIALD and found 6 potential m6A methylation sites (including 5 “RRACH” motif and 1 “ACAGA” motif). We constructed luciferase reporter vectors containing point mutations of these sites (Fig. S4), and the luciferase activity assay showed that mutations in these potential m6A sites did not alter the downregulation of luciferase activity caused by METTL16 compared with the wild-type TIALD sequence, suggesting that METTL16 does not bind to a specific region in TIALD, which was also confirm in Fig. 6F. We speculate that METTL16 binds to TIALD by recognizing its RNA secondary structure, and the mutation of the methylation site in the TIRLAD sequence does not affect its secondary structure, so METTL16 can still bind to these mutants. A recent study showed that METTL16 binds to >4000 mRNA transcripts, the majority of which do not have METTL16-mediated m6A modifications [26], which is coincides with our guess.

Taken together, these data revealed that METTL16, ALKBH5, YTHDF2/YTHDC1 were involved in m6A install, remove and recognize of TIALD, respectively, while m6A-modification decrease the stability of TIALD, thus leading to downregulation of TIALD expression in HCC.

### TIALD physically interacted with AURKA

**Fig. 5:**
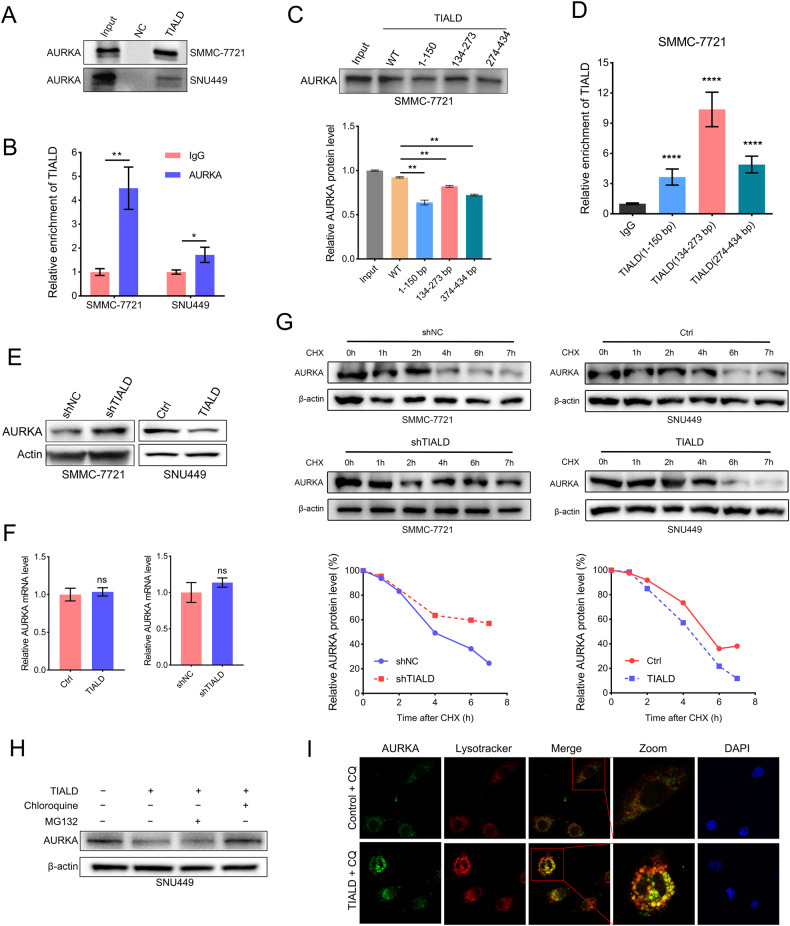
TIALD down-regulated AURKA expression via lysosomal degradation. **A** Western blot showing AURKA interaction with TIALD. RNA pull-down assay was performed in SMMC-7721 and SNU449 cells using biotin-labeled TIALD RNA probe transcribed in vitro, and detected using western blots. **B** RIP-qPCR analysis of the interaction of AURKA and TIALD in SMMC-7721 and SUN449 cells. Enrichment of TIALD with AURKA antibody and IgG control was measured by qPCR. **C** RNA pulldown assays were performed with a series of truncated TIALD variants and followed by immunoblotting with the AURKA antibody. **D** RIP-qPCR analysis were performed using AURKA antibody and IgG control, and enrichment of different TIALD variants was detected by qPCR. **E** The expression of AURKA in TIALD over-expression SNU449 cells and TIALD knock-down SMMC-7721 cells was detected by western blot. **F** The mRNA level of AURKA in TIALD over-expression SNU449 cells and TIALD knock-down SMMC-7721 cells was detected by qPCR. **G** Effect of CHX on AURKA degradation. TIALD over-expression SNU449 cells and TIALD knock-down SMMC-7721 cells were treated with CHX (100 μg/ml) for the indicated times. The expression of AURKA was determined by western blotting and the levels of AURKA protein were quantified by densitometry. **H** TIALD induced AURKA lysosome degradation. TIALD was over-expressed in SNU449 cells and treated with MG132 (20 μM, 3 h) or chloroquine (50 μm, 12 h). The expression of AURKA was analyzed by western blot. **I** TIALD triggers AURKA lysosome localization. TIALD over-expressed SNU449 cells were immune-stained using AURKA antibody followed by Alexa fluor 488 conjugated secondary antibody. Lysosome was marked using Lysotracker. Stained cells were visualized under confocal microscope.

Protein-lncRNA interactions are key aspects of many cellular processes via regulating RNA splicing, transport, stability, translation and lncRNAs often exert their functions by binding one or more proteins [27]. To further investigate the mechanism by which TIALD inhibits HCC metastasis, we further focused our attention on proteins identified by RNA pull down/MS (Fig. 3A). Interestingly, a well-known oncoprotein AURKA, which has been demonstrated to promote metastasis of cancer cells through activating EMT in several cancer types [28, 29] was identified by mass spectrometry as a TIALD binding candidate proteins. Given the important role of AURKA in tumor progression, we speculate that TIALD exerts the function of inhibiting metastasis via binding to AURKA. We then used RNA pull down combined with western blot assays to further verify the interaction between AURKA and TIALD. The results showed that AURKA was specifically gathered by the probes targeting TIALD both in SMMC-7721 and SNU449 cells (Fig. 5A). Parallelly, RIP-qRT-PCR assays demonstrated that tremendous enrichment of TIALD in AURKA immunoprecipitates compared with IgG pellets (Fig. 5B). We further performed deletion-mapping experiments to determine whether AURKA interacts with a specific region of TIALD. Three fragments of TIALD (1–150 bp, 134–273 bp and 274–434 bp) were synthetized to repeat RNA pull down. Unexpectedly, western blot assay showed that all TIALD fragments can be interacted with AURKA, among which fragment 134–273 bp seemingly binding strongest with AURKA (Fig. 5C). RIP-qRP-PCR assays also confirmed that the above three fragments of TIALD were enriched in the AURKA immunoprecipitates and fragment 134–273 bp enriched the most significant (Fig. 5D). These results solidly validated the interaction between TIALD and AURKA.

### TIALD down-regulates AURKA expression via lysosomal degradation

Next, we investigated the effects of TIALD on the expression of AURKA. qRT-PCR and western blot assays showed that TIALD decreased the expression of AURKA at protein level but not mRNA level (Fig. 5E and F) indicating that TIALD might affect the stability of AURKA rather than its transcription and translation. Thereafter, we used protein synthesis inhibitor cycloheximide (CHX) to inhibit protein translation and investigated the stability of AURKA in the cells with the background of TIALD over-expression or knock down. The results indicated that knockdown of TIALD remarkably increased the stability of AURKA. Whereas, over-expression of TIALD accelerates the degradation of AURKA (Fig. 5G). Ubiquitin proteasome pathway and lysosome pathway are the two most important pathways for protein degradation. To determine which pathway is involved in TIALD-promoted AURKA degradation, proteasome inhibitor MG132 and lysosomal inhibitor chloroquine respectively were applied to treat the SNU449 cells with or without TIALD over-expression. Western blot assay showed that protein level of AURKA was maintained in the presence of chloroquine but not MG132 (Fig. 5H). We also re-validated the process of AURKA degradation in C3A cell lines and further treated the cells with Alisertib (Fig. S5). The results were similar with SNU449 cells. MG132 appeared to partially inhibit the degradation of TIALD in C3A, while not as effectively as chloroquine and Alisertib did not appear to interfere with the effect of chloroquine. Additionally, the ubiquitination of AURKA was not detected with or without over-expression of TIALD in the ubiquitination assay (Fig. S6). Confocal images suggested that AURKA colocalized with lysosomes that were labeled by LysoTracker in the cells with TIALD over-expression, indicating that TIALD promotes lysosomal localization of AURKA (Fig. 5I). These data clearly revealed that TIALD interacted with AURKA and mediated its lysosome-dependent degradation. Numerous studies have shown inhibition of protein degradation could contribute to the elevated levels of AURKA expression in cancer tissues, but the current reports on the AURKA degradation pathway focus on the Ubiquitin-proteosomal degradation pathway. In our study, the lysosomal degradation of AURKA was discovered for the first time and it may also provide a new explanation for the molecular mechanism of high expression of AURKA in cancers.

### AURKA mediated the function of TIALD in metastasis inhibition

**Fig. 6:**
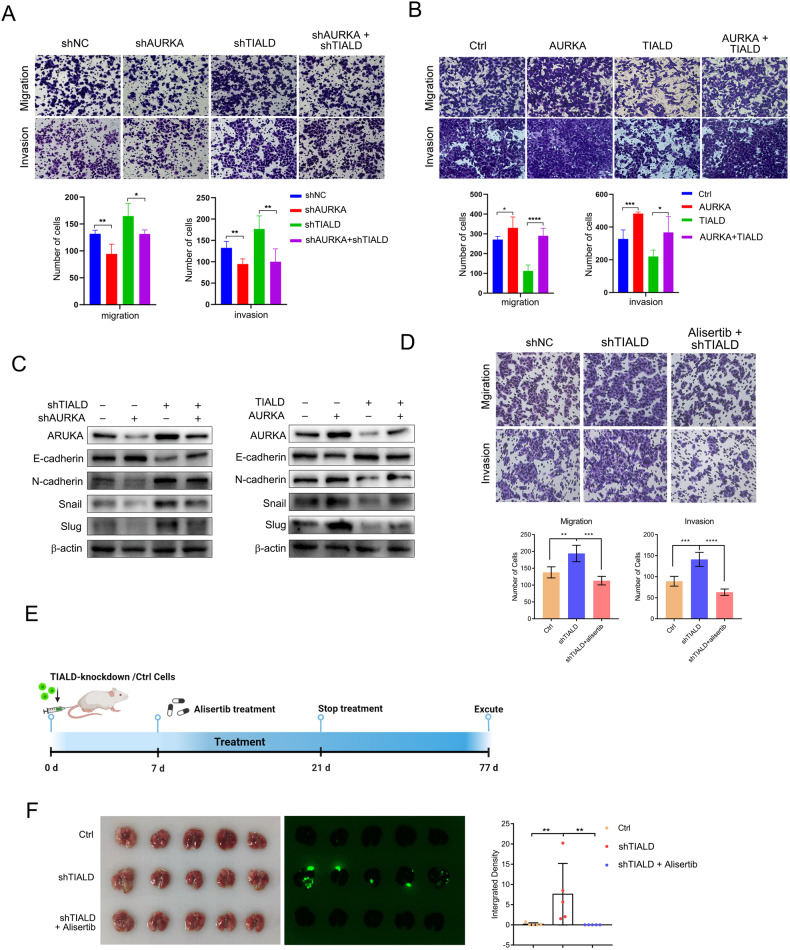
AURKA mediated the function of TIALD in metastasis inhibition. **A** Representative images and quantification results of transwell cell migration and invasion assay in SMMC-7721 cells stably transfected with shTIALD, shAURKA, and co-transfected shTIALD and shAURKA. **B** Representative images and quantification results of transwell cell migration and invasion assay in SNU449 cells stably transfected with TIALD, AURKA, and co-transfected TIALD and AURKA. **C** Western blotting analysis of EMT-associated markers in SMMC-7721 and SNU449 cells as indicated. **D** Representative images and quantification results of transwell cell migration and invasion assay in SMMC-7721 cells stably transfected with shTIALD and treated with Alisertib (10 μM, 12 h). **E**, **F** Representative images and quantification data of metastatic nodules in the lung tissues of mice. Stable cell lines of TIALD knockdown were injected by tail vein in B-NDG mice and treated with Alisertib as indicated.

To verify the ability of TIALD to suppress HCC metastasis in an AURKA-dependent manner, TIALD stably knockdown, over-expression cells or their control cells were transiently transfected by sh-AURKA vector, AURKA over-expression vector or their corresponding empty vector respectively. Restorative experiments exhibited that knockdown of AURKA attenuated the role of TIALD knockdown in enhancing migration and invasion of HCC cells (Fig. 6A). Consistently, opposite results were obtained in the cells with TIALD and AURKA co-over-expressions (Fig. 6B). Moreover, AURKA knockdown or over-expression correspondingly reversed the effects of TIALD on the expressions of E-cadherin, N-cadherin, Slug and Snail, indicates that TIALD inhibits EMT of HCC via an AURKA-dependent manner (Fig. 6C).

As a target for cancer therapy, some small molecules targeting AURKA have been discovered. Alisertib (MLN8237) is the first oral selective AURKA inhibitor to enter phase III clinical trial. Unfortunately, it did not show an apparent effect in prolonging the survival of patients with relapsed or refractory peripheral T-cell lymphoma [30]. Since TIALD can promote the degradation of AURKA, we are curious the effects of alisertib on TIALD down-regulated HCC. Next, alisertib was used for treating SMMC-7721 cells with TIALD knockdown. As shown in Fig. 6D, alisertib could remarkably suppress the migration and invasion of HCC cells with TIALD knockdown, but did not affect the cell viabilities. To further validate the anti-metastasis or recurrence abilities of alisertib in HCC, a tail vein injection mouse model was conducted with TIALD knockdown or control SMMC-7721 cells. Alisertib was oral gavage twice a day into the mouse model at a dose of 20 mg/kg for 5 days, after a 2-day interval, continue dosing for another 5 days (Fig. 6E). As shown in Fig. 6F and Fig. S7, TIALD knockdown significantly promoted the formation of lung metastatic nodules. Importantly, alisertib treatment significantly inhibited the formation of metastatic nodules in the lung, and up-regulated E-cadherin and inhibited N-cadherin expression (Fig. S8), again confirming that inhibition of AURKA could significantly reverse the effect of TIALD knockdown on promoting HCC metastasis.

Collectively, these data suggested that TIALD suppresses HCC metastasis through inducing the degradation of AURKA. More importantly, previous studies showing alisertib exhibited remarkable anticancer effects in preclinical studies and in Phase I and II trials [31], and the low risk of side effects, accessibility, and effectiveness of alisertib makes it a new promising anticancer therapy with further mechanistic and clinical studies, and our study shows that Alisertib may be a drug with great therapeutic potential in TIALD low expressed HCC.

### The METTL16/TIALD/AURKA axis contribute to HCC metastasis

**Fig. 7:**
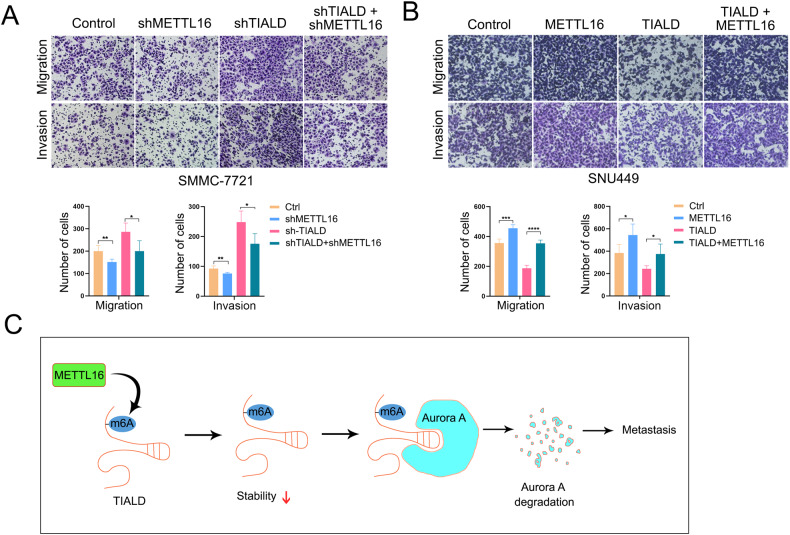
The METTL16/TIALD/AURKA axis contribute to HCC metastasis. **A**, **B** Representative images and quantification results of transwell cell migration and invasion assay in SMMC-7721 cells and SNU449 cells transfected with vector as indicated. **C** Summary of the mechanism by which METTL16 mediated m6A methylation was involved in the downregulation of TIALD by facilitates its RNA degradation, TIALD bind to AURKA and facilitate its degradation through the lysosomal pathway, thus inhibited EMT and metastasis of HCC, lastly, METTL16/TIALD/AURKA axis contributed to HCC metastasis.

Taking together, our findings demonstrate that, on the one hand, METTL16 and its induced m6A methylation are involved in the downregulation of TIALD; and on the other hand, TIALD inhibits the degradation of AURKA, which in turn promotes HCC metastasis. Next, restorative experiments were performed to further confirm the role of METTL16 in regulating HCC metastasis. Transwell assays showed that the migration and invasion of HCC cells were inhibited by knocking down METTL16, which however was reversed by simultaneously down-regulating TIALD (Fig. 7A). Oppositely, over-expression of TIALD could attenuate the roles of METTL16 in promoting migration and invasion of HCC cells (Fig. 7B). Taken together, our finding suggests that, METTL16-mediated m6A modification of lncRNA TIALD accelerated the degradation of TIALD. Then, TIALD inhibited HCC metastasis and EMT through inducing the degradation of AURKA (Fig. 7C).

## Discussion

N6-methyladenosine (m6A) is the most prevalent epigenetic modification of RNA. This biochemical process is proved to play important roles in various physiological and pathological bioprocesses through controlling RNA splicing, translation and stability [32, 33]. Increasing evidences show that m6A methylation modification of noncoding RNAs (ncRNAs) acts an essential role in regulating cell differentiation, angiogenesis, carcinogenesis, metastasis and immune response [34]. Studies have indicated that m6A may contribute to both carcinogenesis or tumor-suppressing via up or downregulation of vital components of cellular signaling. m6A in LncRNA *NEAT1* was shown to exhibit oncogenic function in prostate cancer progression [35], METTL14-induced m6A process suppressed XIST expression, and suppresses proliferation and metastasis of colorectal cancer [36]. Here we show that, METTL16-induced m6A modification in lncRNA TIALD accelerated its degradation, finally contribute to HCC metastasis via inducing AURKA lysosomal degradation.

Studies indicate that m6A modification can act as a structural “switch” to change the conformation of lncRNA, or regulating transcription and stability of lncRNAs. m6A modification can increase RP11 expression in CRC cells by increasing RP11 nuclear accumulation [2], while METTL14-induced m6A process suppressed XIST expression through YTHDF2-dependent RNA degradation [36]. These studies indicated that m6A modification can both up-regulate or down-regulate the expression of target lncRNAs. In our study, m6A writer METTL16, m6A Erasers ALKBH5 and M6A Readers YTHDF2, YTHDC1 were participated in the install, remove and recognize of TIALD m6A modification, and m6a modification results in downregulation of TIALD.

Numerous studies indicated that many m6A modifications are installed by the METTL3–METTL14 complex. More recently, another m6A methyltransferase, METTL16 has now been shown to bind and methylate RNAs such as MAT2A pre-mRNA [4, 37], snRNA [4] and LncRNA MALAT1 [5]. However, studies on METTL16-mediated RNA m6A methylation are still minimal. Whether METTL16 methylates a large set of transcripts, similar to METTL3 and METTL14, remains unclear. Our results show that METTL16 is able to induce m6A methylation in TIALD. We mutated potential m6A methylation sites (including RRACH motif and ACAGA motif) in the TIALD sequence. Unfortunately, the luciferase reporter assay indicated that even if the m6A sites were mutated one by one, METTL16 was still able to bind to TIALD. A recently published study indicated that METTL16 exerts both methyltransferase activity-dependent and -independent functions in gene regulation. METTL16 may act as a translation facilitator through recruitment eIF3a/b and ribosomal RNAs [26]. This study, together with our results, demonstrates that METTL16 not only binds to the m6a motif, but also to unmethylated transcripts, possibly through specific RNA secondary structures.

AURKA (Aurora-A) is a member of Aurora kinase family involved in the regulation of mitosis, and is reported to be over-expressed in many human cancers including HCC [14, 38]. Upregulation of AURKA was correlated with poor outcomes in HCC patients [39]. Functionally, it plays multiple roles in regulating cancer cell proliferation, apoptosis, metastasis, stemness and genomic instability [13, 40, 41]. In HCC, AURKA promotes cancer metastasis and cancer stem cell properties [42]. The regulation of AURKA expression mainly includes gene amplification, transcriptional upregulation or protein stabilization [14]. Our study indicates that lncRNA is also involved in the regulation of AURKA protein stability, as we show that TIALD promotes the degradation of AURKA. Interestingly, in previous studies, ARUKA was almost degraded by ubiquitin-proteasome pathway. For example, SMAD4 and SOCS2-AS1 may bound to AURKA and increased its degradation through the ubiquitin-proteasome pathway [19, 43]. The study of AURKA degradation by lysosome has not been reported. Our study shows for the first time that AURKA can be degraded in a lysosome-dependent manner by via binding to lncRNA. Our findings expand our understanding of the AURKA degradation pathway, and provides a new theoretical basis for the cancer therapy targeting AURKA, but more studies are needed on how TIALD directs its targeting to lysosomes by binding to AURKA.

The AURKA inhibitor alisertib has shown to have great antitumor activity both alone and in combination with other conventional therapies in both preclinical and clinical studies [31]. However, its clinical efficacy varies across different cancer types. Therefore, deciphering the underlying oncogenic mechanisms of AURKA in different malignancies is crucial to the use of AURKA inhibitors for the control of disease progression. HCC has a very poor clinical prognosis due to its high recurrence and metastasis rate. For advanced HCC, Sorafenib was the first systemic therapy approved drug. New drugs—lenvatinib in the frontline and regorafenib, cabozantinib, and ramucirumab in the second line—have also been demonstrated to improve clinical outcomes, but the median overall survival of these treatments still remains ~1-year, therapeutic breakthroughs are still needed. A recent study showed that alisertib-lenvatinib combination contributes to the anti-tumor activity in HCC cells and xenograft tumors model [44]. The role of AURKA inhibitor in the treatment of HCC still needs more studies, including exploring more combined treatment methods and screening potential benefit patient groups, which will speed up the clinical promotion of alisertib in the treatment of HCC, and also provide more treatment options for advanced HCC. Our findings provide evidence that AURKA inhibitor would be a therapeutic approach for the treatment of advanced HCC and TIALD may be a potential target that could enhance the activity of alisertib, that would provide a rationale for drug combination to achieve better therapeutic outcome.

In summary, our study revealed that lncRNA TIALD acted as a tumor suppressor which was down-regulated via METTL16 mediated m6A methylation, and contributed to HCC metastasis via inducing degradation of AURKA, and AURKA inhibitor alisertib exhibited significant effect of inhibiting metastasis in TIALD knockdown HCC. These findings provide new insights into our understanding of the molecular networks between lncRNA m6A methylation and tumor metastasis, and proposed therapeutic and prognostic targets for the clinical treatment of HCC.

## Materials and methods

### Clinical specimens

For RNA-seq assay, 8 freshly frozen HCC tumor and their paired adjacent non-tumor tissues were obtained from the bio-bank of Mengchao Hepatobiliary Hospital of Fujian Medical University. For validation and prognosis analysis, another sample cohort including HCC tumor and non-tumor tissues were collected from 110 HCC patients with clinical characteristics and long-term follow-up data at Mengchao Hepatobiliary Hospital of Fujian Medical University from January 2016 to December 2020. All the enrolled patients were diagnosed with HCC and without receiving any interventional treatment prior to sample collection. This study was approved by Mengchao Hepatobiliary Hospital Medical Ethics Committee of Fujian Medical University (2020_090_01). Informed consents were received from each participant before surgery.

### Cell lines, cell culture and reagents

Human HCC cell lines SNU-449, SK-Hep-1, C3A and Hep3B were purchased from American Type Culture Collection (ATCC, VA, USA). SMMC-7721 cell line as a kindly gift was obtained from Professor Wang Hongyang at the Second Military Medical University (China), which has been used in previous study [45]. These cell lines were characterized by short tandem repeat (STR) analysis by the third-party biology services (Genewiz, Suzhou, China). SNU-449 cells were maintained with RPMI-1640 (Gibco, USA), SK-Hep-1, C3A and Hep3B cells were cultured with MEM (Gibco, USA) and SMMC-7721 cells were maintained with DMEM. All mediums were supplemented with 10% FBS, 100 IU penicillin and 100 mg/mL streptomycin (Gibco, USA).

Actinomycin D (2 μg/mL)、MG132 (100 μM)、Cycloheximide (CHX, 100 μg/mL) and Chloroquine (50 μM) were purchased from Selleck company. Lysotracker (Lysotracker Deep Red, L12492) was from Thermo Fisher.

### Transient transfection and construction of stably modified cell lines

The full-length TIALD sequence was synthesized by General Biol (China) and cloned into a lentiviral vector to construct a TIALD overexpression vector, pCDH-TIALD-puro-copGFP. To knock down the target genes, the same cDNA oligonucleotides with the shRNAs against TIALD, METTL16, YTHDF2, YTHDC1 were synthesized by Sangon Biotech (China) respectively. The sequences were listed in Supplementary Table S2. After annealed, double-strand oligonucleotides were inserted into pLKO.1 vector (Addgene). For transient transfection, cells were transfected using Lipofectamine 3000 (Thermo, USA) and harvested for the following assays after 48 h of transfection.

To construct stably modified cell lines, plasmids pCDH-TIALD-puro-copGFP and pLKO.1-shTIALD-EGFP were firstly applied to generated lentiviruses (LV-TIALD-GFP and LV-shTIALD-GFP) as previously described respectively [46]. Afterwards, lentiviral particles were concentrated by Optima XPN-100 Ultracentrifuge (Beckman Coulter, USA) at 15000 g for 2 h under vacuum at 4 °C, and then the concentrated lentiviral particles were resuspended by 500 μL PBS. SNU-449 and SMMC-7721 cells with 50% cell density in 6 well cell culture plates were infected by 100 μL lentivirus for 2 days in the presence of polybrene (1 μg/mL, Santa Cruz Biotechnology, USA). The infected cell lines were further selected by puromycin (2 μg/ml) and verified by qRT-PCR.

### RNA-seq assay

HCC tumor tissues paired with their adjacent non-tumor tissues from 8 patients were used for RNA-seq assay. Sequencing libraries were established by VAHTS® Universal V6 RNA-seq Library Prep Kit from Illumina (Vazyme, China) following the manufacturer’s instruction. Whole transcriptome sequencing (paired end, 150 bp) was conducted on the libraries using Illumina HiSeq X10 at Annoroad Gene Tech. Co., Ltd (Beijing, China). The Illumina short reads were aligned to the human reference genome (Ensembl GRCh38) with annotations (GENCODE GRCh38 v32) using STAR (v2.6.0) on the 2-pass mode, followed by quantification of annotated transcripts using RSEM (v1.3.1). Differential expression analysis was performed using R software. Genes with |log_2_[fold change]| > 1 and FDR < 0.05 were considered to be differentially expressed in HCC tissues and matched para-tumor tissues.

### qRT-PCR assay

Total RNA was extracted from cell lines using TransZol Up RNA kit (Code#ER501-01-V2, TransGen, Beijing, China). Nanodrop 2000 (Thermo Fisher, USA) was used to detect the RNA concentration. cDNA was synthesized by using the Transcriptor Frist Strand cDNA Synthesis Kit (5081955001, Roche, Basel, Switzerland) with random primers. Quantitative PCR was performed with SYBR Green qPCR Master mix (DBI-2233, DBI, Ludwigshafen, Germany). Human 18 S rRNA was used as endogenous control. The PCR amplification was subsequently performed: 95 °C for 5 min, followed by 95 °C for 10 s, 60 °C for 30 s. The relative expression of RNA was calculated by the 2^−ΔΔCt^ method [47]. All qPCR primer sequences were listed in supplementary Table S1.

### In vitro cell migration and invasion assay

The migration and invasion of SNU-449 or SMMC-7721 cells were examined by transwell system (8 mm pore, Corning), according to the manufacturer’s instruction as previously described [48]. Wound healing assay was performed to examine the mobility of HCC cells either; accordingly, 7 × 10^4^ cells in 70 μl medium were seeded into each side of the insertion (Ibidi, Gräfelfing, Germany); after 12 h of incubation, the insertion was removed and the cells were washed twice prior to further culture. Then, the images were captured at 0, 6, 12, 24, and 36 h.

### Animal studies

All animal experiments were approved by the Animal Experimentation Ethics Committee of Mengchao Hepatobiliary Hospital of Fujian Medical University. To evaluate the effects of TIALD on the metastasis of HCC cells, 22 of 6-week-old female B-NDG mice (NOD.CB17-Prkdcscid Il2rgtm1/Bcgen) were purchased from Biocytogen (China) and kept in a specific pathogen-free barrier. 1 × 10^6^ of TIALD stable overexpression, knockdown or control cells were resuspended in 100 μl PBS (magnesium and calcium free) and injected into the mice via tail vein. The mice were euthanized after 6 weeks of injection. Then, the lungs were removed from the mice and visualized by IVIS@ Lumina II system (Caliper Life Sciences, USA). In the therapeutic study, TIALD stable knockdown or control cells were further infected by lentiviruses (Ubi-MCS-firefly_Luciferase-SV40-neomycin, Genechem, China) and selected by G418 (500 μg/mL). 45 B-NDG mice were divided into 3 groups, including the control group (*n* = 15), TIALD knockdown group (*n* = 15) and Alisertib treatment group (*n* = 15). The modified control cells described above were injected into the mice of control group via tail vein, while the modified TIALD knockdown cells were injected into the mice of TIALD knockdown group and Alisertib treatment group. Alisertib (HY-10971, MedChemExpress, USA), an inhibitor of AURKA, which was dissolved in 1% sodium bicarbonate solution with 10% Hydroxypropyl-β-cyclodextrin, was used to inhibit the metastasis of HCC cells promoted by TIALD knockdown. After 1 week of injection, the mice were subjected to 20 mg/mL Alisertib or control treatment via oral gavage twice daily, using a 5 days on/2 days off schedule for 2 weeks. The mice were imaged once a week by IVIS@Lumina II system (Caliper Life Sciences, USA). After 56 days of treatment, the mice were euthanized. Each lung was subsequently split into two fractions after visualization. Total RNA was extracted from one fraction for qRT-PCR assay, the other fraction was used for pathological analysis.

### Dual-luciferase reporter assay

The dual-luciferase reporter assay was performed following the manufacturer’s instructions. 293 T cells were transient transfected with pmirGLO-luciferase vector fused or not fused to the wild type or mutated TIALD. METTL16 or empty vectors were co-transfected. After transfecting for 48 h, the 293 T cells were lysed and the luciferase activity was detected by using the TransDetect^®^ Double-Luciferase Reporter Assay Kit (Code#FR20102, TransGen, Beijing, China). Firefly luciferase activity and renilla luciferase activity were measured by SpectraMax M5e Multi-Mode Microplate Reader (Molecular Devices, USA). Firefly luciferase activity was normalized by renilla luciferase values. All experiments were performed in triplicate.

### RNA pull-down combined with mass spectrometry analysis

Full length sequence of TIALD was in vitro transcribed with AmpliScribe™ T7 High Yield Transcription Kit (Cat.AS3107, Lucigen, USA) and biotinylated with Pierce™ RNA 3’ End Biotinylation Kit (20160, Thermo, USA) according to the manufactures’ protocol. The RNA products were purified with a RNeasy Mini Kit (Qiagen, USA). Then, 5 μg biotinylated RNAs were denatured for 5 min at 65 °C in Binding buffer (10 mM Tris HCl pH7.5, 10 mM MgCl_2_, 100 mM NH_4_Cl), cooled down to room temperature. Afterwards, 1 × 10^7^ SMMC-7721 cells were lysed with lysis buffer (50 mM Tris-HCl, pH 7.5; 150 mM NaCl; 1% Triton X-100; 0.1% SDS and 1% protease inhibitor cocktails). The folded RNA was subsequently co-incubated with streptavidin beads and cell lysates for 4 h at 4 °C. After wash for 6 times, the beads were boiled with 40 μl of 1× SDS loading buffer for 10 min at 100 °C. The proteins interacting with TIALD were analyzed by mass spectrometry or western blot. For mass spectrometry, the beads were incubated with 50 μL elution buffer at 37 °C for 30 min Afterwards, the proteins were reduced by 8 mM DTT at 55 °C for 30 min, alkylated by 50 mM IAA in dark for 30 min, and digested to peptides by trypsin at 37 °C for 18 h. Then, the peptides were dried by vacuum rotary dryer at 32 °C. 1 μg peptides dissolved by 4 μL 0.1% FA solution were added to the nano Acquity UPLC (Waters, USA) and analyzed by Thermo Q Exactive Plus (Thermo Fisher, USA) in a DDA mode. All the data were searched by Maxquant v4.2 and matched with the database supplied by The Universal Protein Resource (http://www.uniprot.org/uniprot).

### RNA immunoprecipitation (RIP)-qPCR and meRIP-qPCR assay

RIP was performed using the Magna RIPTM RNA-binding Protein Immunoprecipitation Kit (Millipore, USA) following the manufacturer’s instruction. Briefly, the lysates of 2*10^7^ SMMC-7721 or SNU-449 cells were incubated with beads pre-coated with IgG or antibody against METTL16 (#87538, CST, USA) or AURKA (ab13824, Abcam, USA) overnight at 4 °C in the presence of 2U/ml RNasin (Promega, USA). The incubated beads were washed with 500 μL RIP wash buffer by transient vortex for six times. Afterwards, the beads were resuspended in proteinase K buffer including 10 mg/mL proteinase K and 10% SDS, and incubated at 55 °C for 30 min. The immunoprecipitated RNAs were then extracted with the mixture (phenol: chloroform: isoamylol = 25:24:1) by centrifuging at 14,000 rpm for 10 min at room temperature, and purified with ethanol including 0.5 M NaCl and 50 μg/mL GlycoBlue Coprecipitant (AM9516, Thermo Fisher, USA) at −80 °C overnight. The precipitates of RNA were centrifuged at 14,000 rpm for 30 min at 4 °C, resuspended in 10 μL RNase-free water and further detected by qRT-PCR. The meRIP assay was performed as RIP assay. The differences were that whether the cells were overexpressed METTL16 or empty vector and the beads were pre-coated with antibody against m6A modification (202003, 5 μg, SYSY, Germany).

### RNA stability

SNU-449 cells with METTL16 knock down, SMMC-7721 cells with METTL16 overexpression and their control cells were treated with 2 μg/ml actinomycin D (Act-D, Sigma-Aldrich, USA) to measure the stability of RNA, respectively. After the incubation for the indicated time, total RNAs were extracted from the treated cells for qRT-PCR. The expressions of TIALD were normalized by 18 s rRNA.

### Protein stability

To determine the stability of AURKA, the translations of genes were stopped by cycloheximide (CHX, 100 μg/ml, Selleck, China) in SNU-449 cells with TIALD overexpression and SMMC-7721 cells with TIALD knockdown and their control cells. Cells were lysed by RIPA buffer supplemented with 1% protease inhibitor cocktails at the indicated time periods. And the expressions of AURKA were detected by western-blot assay.

### RNA fluorescence in situ (FISH)

Fluorescence in situ hybridization (FISH) was performed as previously described [23]. Briefly, modified SNU-449, SMMC-7721 and control cells grown on the slides were washed three times with PBS and fixed with 4% paraformaldehyde for 15 min. After treatment with 0.5% Triton X-100 for 5 min, the slides were pre-incubated with prehybridization buffer at 37 °C for 30 min and then hybridized with digoxin-conjugated probe at 37 °C overnight. After blocking with 5% BSA in PBS for 30 min and washing, the slides were incubated with anti-digoxin antibody conjugated with HRP at 37 °C for 1 h and washed three times, then the slides were further incubated with cy3-Tyramide at room temperature for 5 min and washed again. DAPI was used for labeling nuclear DNA. Afterwards, the slides were visualized under a confocal microscope (Zeiss LSM780, Germany). The probe sequences were listed in supplementary Table S3.

### Immunohistochemistry (IHC) analysis

IHC analysis was performed as previously described [49] with slight modification. Briefly, the slides were deparaffinized, rehydrated, and then subjected to high-pressure for antigen retrieval in EDTA antigen retrieval buffer. 3% hydrogen peroxide was conducted to inactivate endogenous peroxidase activity. After blocking with 5% BSA for 20 min, the slides were incubated with antibodies against E-cadherin (#3195,1:200 dilution, CST, USA), N-cadherin (#13116, 1:125 dilution, CST, USA), AURKA (ab52973, 1:500 dilution, Abcam, USA). After incubated with antibodies overnight at 4 °C, the slides were washed three times with PBS and incubated with secondary antibody from Elivision^TM^ plus Polyer HRP (Mouse/Rabbit) IHC Kit (Maixin Bio, China) for 30 min. After washing thoroughly three times, the slides were incubated with DAB reaction buffer for 3–5 min and the brown precipitate showed a positive signal, and then the slides were counterstained with hematoxylin. IHC staining was visualized using an Olympus BX40 microscope (Olympus Co., Japan).

### Western-blot assay

Western blot assay was performed as previous description [48] with slight modification. Briefly, proteins were extracted by RIPA buffer (Beyotime, China) in the presence of 1% protease inhibitor cocktails and phosphostop (Roche, Germany). BCA Protein Assay Kit (Transgene, China) was subsequently conducted to quantify the concentrations of protein following the manufacturer’s instructions. Equal amount of each sample was separated by 10% SDS-PAGE and transferred onto NC membrane (PALL Corporation, USA). After blocking with 5% skim milk for 1 h at room temperature, the membranes were probed with primary antibodies respectively at 4 °C overnight. Afterwards, the membranes were incubated with horseradish peroxidase-conjugated goat anti-rabbit (2000 folds dilution, CST, USA) or goat anti-mouse (2000 folds dilution, CST, USA) secondary antibody. After wash 5 times with TBST, the blots were detected using ChemiDoc MP Imaging System (BIO-RAD, USA). Blots were quantified by measuring the densitometry. All experiments were repeated at least twice. The primary antibodies against E-cadherin (1000 folds dilution), N-cadherin (1000 folds dilution), Snail (#3879, 1000 folds dilution), Slug (#9585, 1000 folds dilution) and METTL16 (1000 folds dilution) were purchased from cell signaling technology (CST, USA). The primary antibody against AURKA (1000 folds dilution) and the horseradish peroxidase-conjugated antibody against β-actin (5000 folds dilution) were obtained from Abcam, USA.

### Statistical analysis

Prism statistical software (GraphPad v8.21, USA) and SPSS statistics (v25.0, SPSS Inc., USA) were used for data analysis. All data were presented as mean ± SD from at least three independent experiments with duplicate or triplicate samples. Student *t*-test or *x*^2-p^test was applied to compare the difference between two groups. Comparisons in three or more groups were analyzed by One-way ANOVA. Kaplan–Meier curves with log-rank test were used for survival analysis. Cox proportional hazards models were used for evaluating the correlations between variables and prognosis of HCC patients. *P*-value < 0.05 was considered to be statistically significant.

### Supplementary information

supplementary information

STR profile report

Original Data File

## Data Availability

All data generated or analyzed during this study are included either in this article or in the supplementary information files.
