# Supplementary material for: LncRNA TIALD contributes to hepatocellular carcinoma metastasis via inducing AURKA lysosomal degradation
Source: Cell Death Discov. 2023 Aug 26;9:316. doi: 10.1038/s41420-023-01620-w (PMC10541412; doi:10.1038/s41420-023-01620-w)
Supplement: Supplementary file 1 — supplementary information [file 41420_2023_1620_MOESM1_ESM.docx]

LncRNA TIALD contributes to hepatocellular carcinoma metastasis via inducing AURKA lysosomal degradation

**Supplementary information**

**Supplementary Figure**


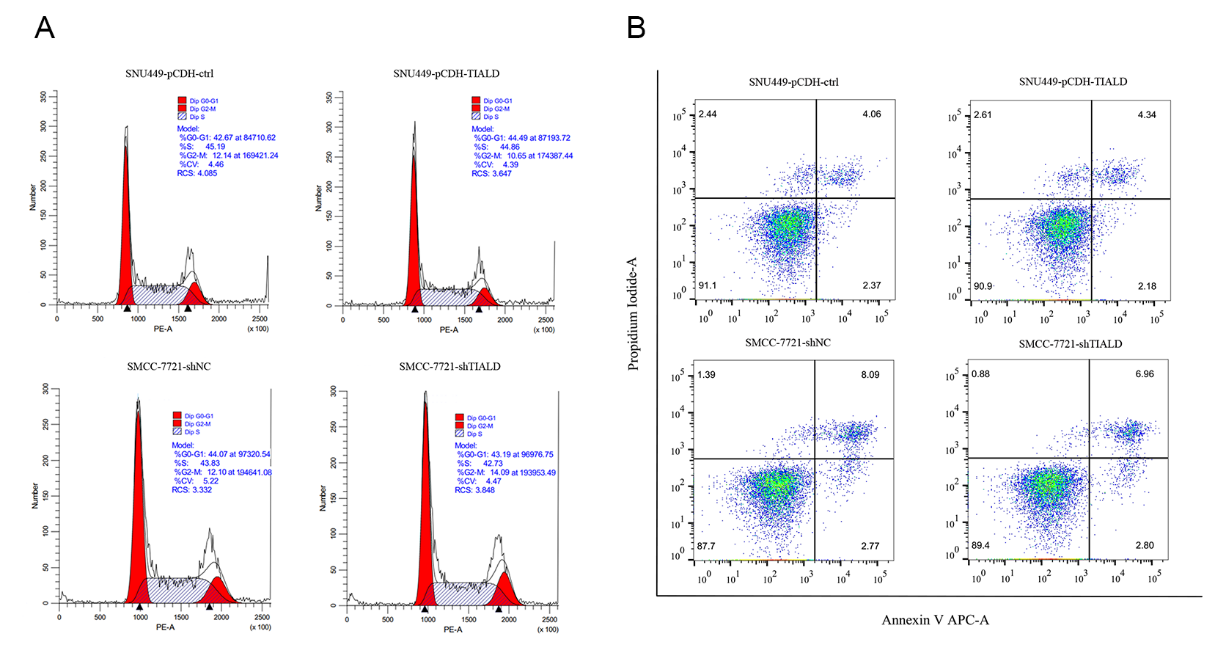


**Supplementary Figure S1**. Cell cycle and cell apoptosis affected by TIALD. (A) Cell cycle assay was performed in TIALD overexpression SNU449 cells and TIALD knock-down SMMC-7721 cells and detected by Cell Cycle and Apoptosis Analysis Kit (Beyotime Biotechnology, China). (B) Cell apoptosis was performed in the same cells with cell cycle and detected by Annexin V-APC/PI Apoptosis Detection Kit (Keygen Biotech, China).


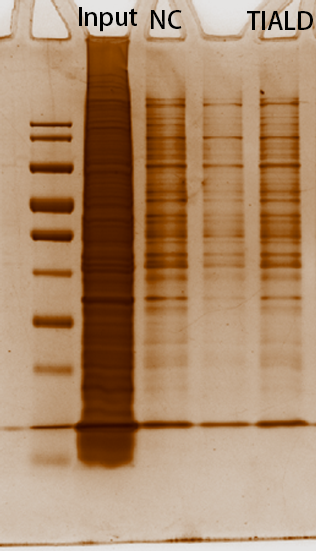


**Supplementary Figure S2**. Silver stain of the TIALD IP gel. NC group was negative control and it was used the poly(A)25 RNA from Thermo Fisher Scientific (supplied with the RNA pull-down kit).


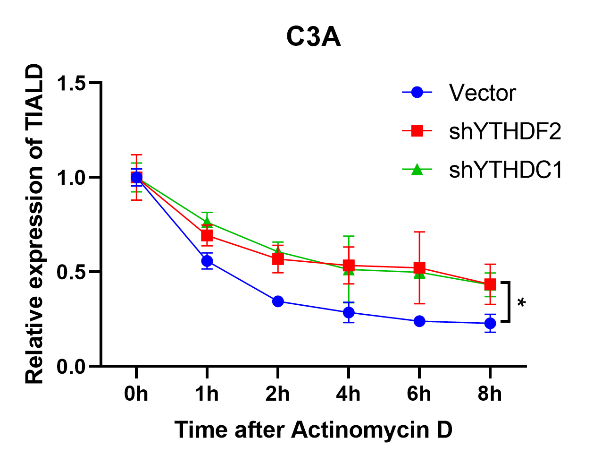

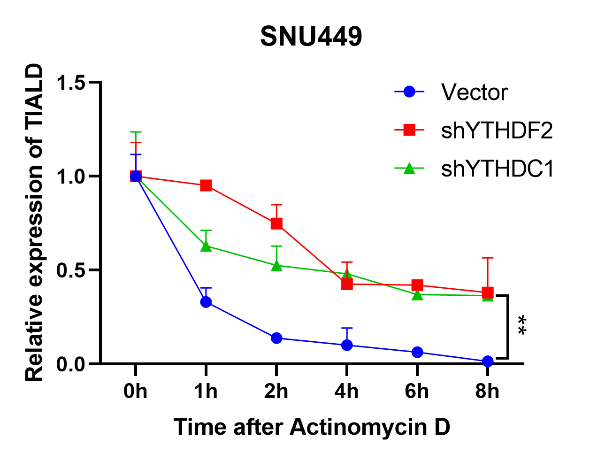


**Supplementary Figure S3.** The degradation of TIALD affected by knockdown of YTHDF2 and YTHDC1. Cells were treated with 1 μM actinomycin D.


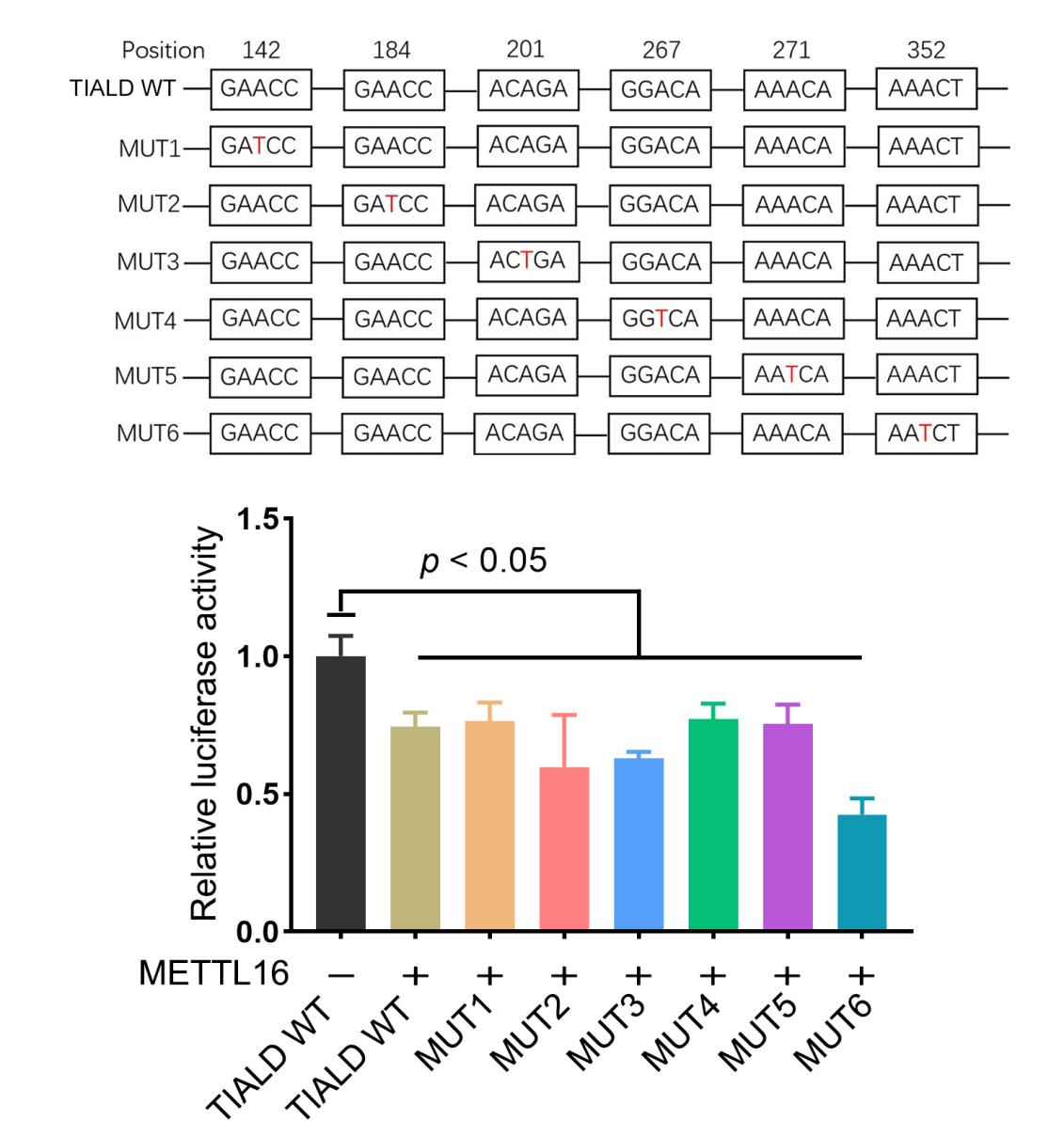


**Supplementary Figure S4.** Luciferase reporter assay to validate the interaction between MTTL16 and TIALD point mutations. Wild-type TIALD and with a mutation at the m6A consensus sequence were cloned into a luciferase reporter. Relative luciferase activity of the wild-type and 6 mutant TIALD reporter vectors catalyzed by METTL16.


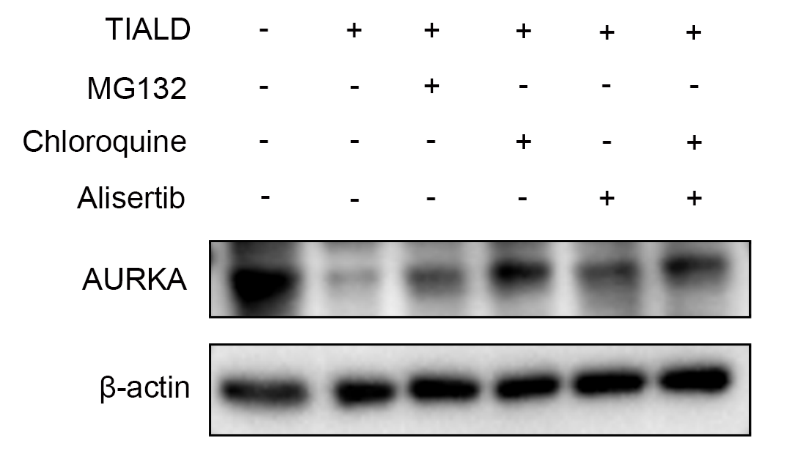


**Supplementary Figure S5.** C3A cells were transfected with TIALD or control vector and treated with MG132 (100 μM, 6h) or chloroquine (100 μM, 6h) or Alisertib (10 μM, 6h), respectively.

**
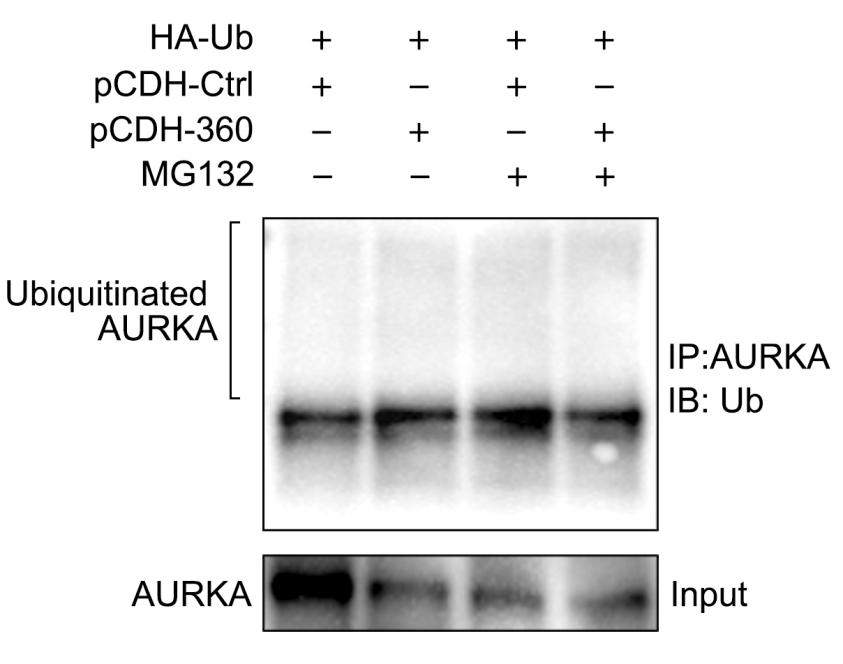
**

**Supplementary Figure S6.** Effects of TIALD on AURKA ubiquitination.

**
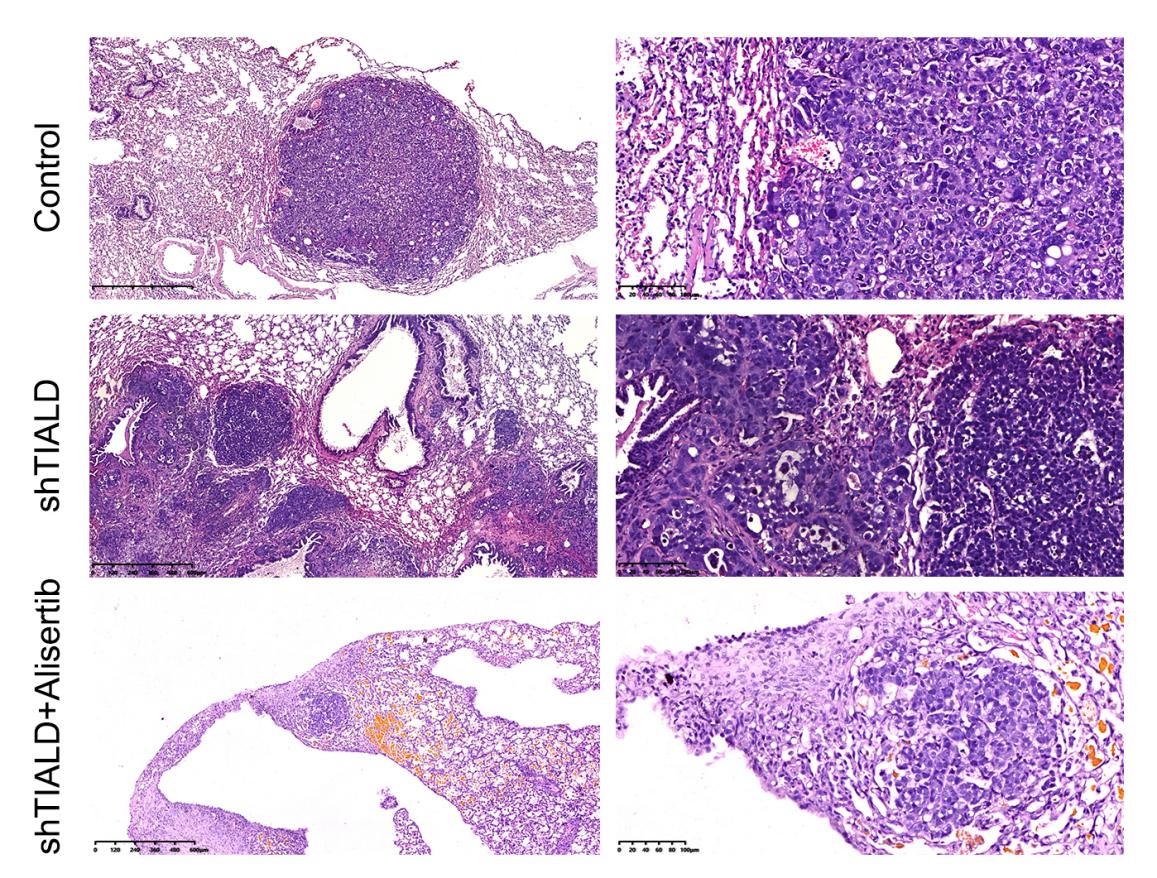
**

**Supplementary Figure S7.** Representative images of lung metastatic nodules stained with H&E.

**
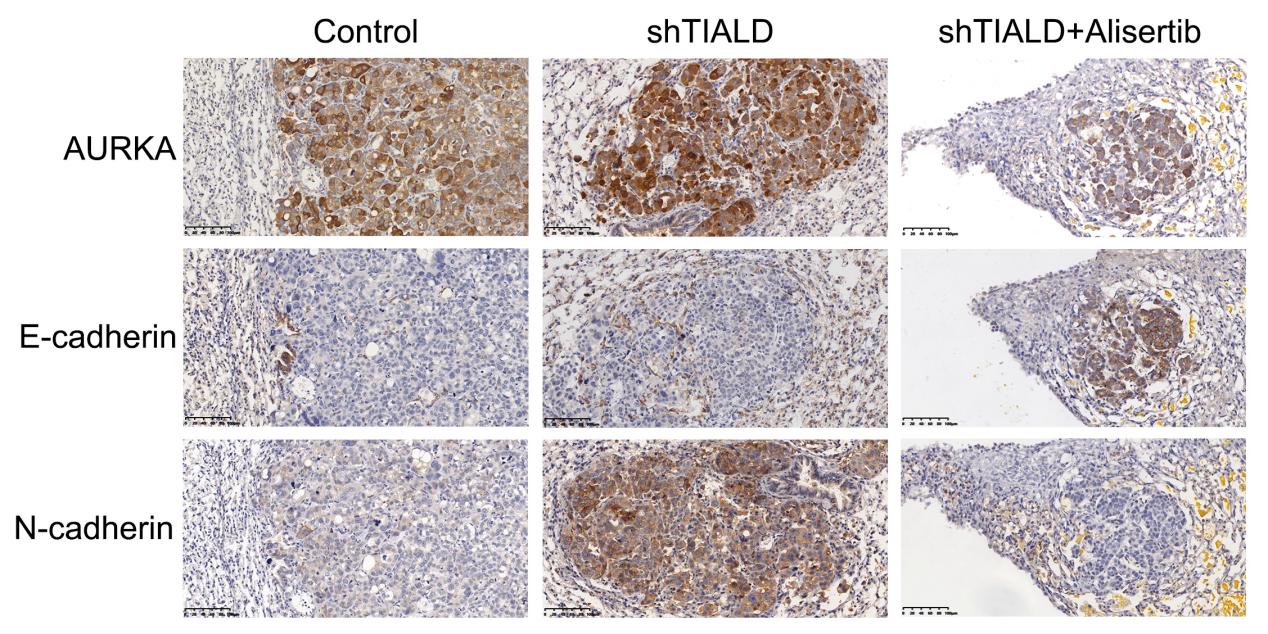
**

**Supplementary Figure S8**. Representative images of IHC staining in lung metastasis nodules of mice.

**Supplementary Table**

Supplementary Table S1. qPCR primer sequences

| Gene symbol | Sequences (from 5’ to 3’) |
| --- | --- |
| TIALD | F: ATCTTCCACCTCGAACGCAA |
|  | R: AGGTGAGGGGACCTAGACTC |
| AURKA | F: GAGGTCCAAAACGTGTTCTCG |
|  | R: ACAGGATGAGGTACACTGGTTG |
| METTL16 | F: CTCTGACGTGTACTCTCCTAAGG |
|  | R: TACCAGCCATTCAAGGTTGCT |
| ALKBH5 | F: CGGCGAAGGCTACACTTACG |
|  | R: CCACCAGCTTTTGGATCACCA |
| YTHDF2 | F: GTTGGTAGCGGGTCCATTACT |
|  | R: GGTCTTCAGTTTAGGTTGCTGT |
| YTHDC1 | F: CTTCTGATGAGCAAGGGAACAA |
|  | R: GGCCTCACTTCGAGTGTCATAA |
| 18S | F: AGAAACGGCTACCACATCC  R: CACCAGACTTGCCCTCCA |

Supplementary Table S2. shRNA sequence

| Gene symbol | Sequences (from 5’ to 3’) |
| --- | --- |
| shTIALD | Top Strand: GATCCCACCGAGGAAACGAAACTAGGAATCAAGAGTTCCTAGTTTCGTTTCCTCTTTTTG |
|  | Bottom Strand: AATTCAAAAAGAGGAAACGAGGAAAAACTCTCTTGATTCCTAGTTTCGTTTCCTCGGTGG |
| shAURKA | Top Strand:  CCGGGATGCCCTGTCTTACTGTCACTCGAGTGACAGTAAGACAGGGCATTTTTTG |
|  | Bottom Strand:  AATTCAAAAAATGCCCTGTCTTACTGTCACTCGAGTGACAGTAAGACAGGGCAT |
| shMETTL16 | Top Strand:  GATCCGCAACAGAAGTGGATGATATGTTCAAGAGACATATCATCCACTTCTGTTGCTTTTTT |
|  | Bottom Strand:  AGCTAAAAAAGCAACAGAAGTGGATGATATGTCTCTTGAACATATCATCCACTTCTGTTGCG |
| shYTHDF2 | Top Strand:  CCGGCGGTCCATTAATAACTATAACCTCGAGGTTATAGTTATTAATGGACCGTTTTTG |
|  | Bottom Strand:  AATTCAAAAACGGTCCATTAATAACTATAACCTCGAGGTTATAGTTATTAATGGACCG |
| shYTHDC1 | Top Strand:  CCGGTGCCTCCAGAGAACCTTATAACTCGAGTTATAAGGTTCTCTGGAGGCA TTTTTG  Bottom Strand:  AATTCAAAAATGCCTCCAGAGAACCTTATAACTCGAGTTATAAGGTTCTCTGGAGGCA |

| Gene symbol | Probe Sequences (from 5’ to 3’) |
| --- | --- |
| TIALD | CAACAAAAACATATAATTCAGCTCCCACCGACCC (ttt CATCATCAT ACATCATCAT) |

Supplementary Table S3. FISH probe sequence

Supplementary Table S4. Univariate and multivariate cox regression analysis affecting overall survival of HCC patients

| **Variable** | **Case number** | **HR** | **95% CI** | ***P* value** |
| --- | --- | --- | --- | --- |
| **Univariate analysis** |  |  |  |  |
| **TIALD (high vs. low)** | 55/55 | 2.026 | 1.053-3.899 | **0.034** |
| **Gender(female vs. male)** | 20/90 | 0.532 | 0.258-1.096 | 0.087 |
| **Age, years (≥55 vs.＜55)** | 51/59 | 0.518 | 0.266-1.010 | 0.054 |
| **Tumor size, cm (≥5 vs.＜5)** | 52/58 | 3.996 | 1.985-8.044 | **<0.001** |
| **Tumor number (multiple vs. single)** | 3/107 | 0.844 | 0.116-6.150 | 0.867 |
| **AFP, ng/mL (≥400 vs.＜400)** | 33/77 | 2.541 | 1 .349-4.788 | **0.004** |
| **Vascular invasion (yes vs. no)** | 55/55 | 4.234 | 2.008-8.925 | **<0.001** |
| **Tumor capsule (complete vs. none)** | 92/18 | 3.830 | 1.956-7.500 | **<0.001** |
| **TNM stage(III-IV vs. I-II)** | 25/85 | 3.845 | 2.020-7.319 | **<0.001** |
| **Tumor differentiation (III-IV vs. I-II)** | 78/32 | 2.871 | 1.202-6.859 | **0.018** |
| **Multivariate analysis** |  |  |  |  |
| **TIALD (high vs. low)** | 55/55 | 2.237 | 1.105-4.528 | **0.025** |
| **Tumor size, cm (≥5 vs.＜5)** | 52/58 | 2.699 | 1.221-5.694 | **0.014** |
| **Vascular invasion (yes vs. no)** | 55/55 | 2.382 | 0.986-5.754 | **0.054** |
| **Tumor capsule (complete vs. none)** | 92/18 | 2.679 | 1.318-5.447 | **0.006** |
