# Supplementary material for: LncRNA TIALD contributes to hepatocellular carcinoma metastasis via inducing AURKA lysosomal degradation
Source: Cell Death Discov. 2023 Aug 26;9:316. doi: 10.1038/s41420-023-01620-w (PMC10541412; doi:10.1038/s41420-023-01620-w)
Supplement: Supplementary file 2 — STR profile report [file 41420_2023_1620_MOESM2_ESM.pdf]

GENETIC TESTING BIOTECHNOLOGY

Cell Line Authentication Service
STR Profile Report

Sample Submitted By: Dr. Yingchao Wang
Mengchao Hepatobiliary Hospital of Fujian Medical University
Email Address: 15060092239@163.com
Sales Order: 221202C
Cell Line Designation: Hep-G2/C3A
Date Sample Received: Dec 2th, 2022
Report Date: Dec 5th, 2022

Methodology: Twenty-one short tandem repeat (STR) loci, one Y-Indel locus plus the Amelogenin locus were amplified using the commercially available SiFaSTR™ 23 plex Kit. The cell line sample was processed using the ABI Prism® 3130 XL Genetic Analyzer. Data were analyzed using GeneMapper® ID v3.2 software (Applied Biosystems). Appropriate positive and negative controls were run and confirmed for each sample submitted.

Data Interpretation: Cell lines were authenticated using Short Tandem Repeat (STR) analysis as described in 2012 in ANSI Standard (ASN-0002) by the ATCC Standards Development Organization (SDO) and in Capes-Davis et al., Match criteria for human cell line authentication: Where do we draw the line? Int J Cancer. 2013;132(11):2510-9.

GTB™ performs STR Profiling following ISO 9001:2008 and ISO/IEC 17025:2005 quality standards.
There are no warranties with respect to the services or results supplied, express or implied, including, without limitation, any implied warranty of merchantability or fitness for a particular purpose. Genetic Testing Biotechnology (GTB) is not liable for any damages or injuries resulting from receipt and/or improper, inappropriate, negligent or other wrongful use of the test results supplied, and/or from misidentification, misrepresentation, or lack of accuracy of those results. Your exclusive remedy against GTB and those supplying materials used in the services for any losses or damage of any kind whatsoever, whether in contract, tort, or otherwise, shall be, at GTB's option, refund of the fee paid for such service or repeat of the service.

NOTE: According to the recommendations of *IJC* on cell line authentication, the report is valid for 3 years since the issue date.

Technical Questions?
GTB Technical Support
+86-512-67486171
service@jsdna.org
Section 505, Yixin BLD
SIP, Suzhou, 215123
Jiangsu, P.R. China

Ordering Questions?
order@jsdna.org
GTB Corporation
+86-512-62806339
Section 303, Yixin BLD
SIP, Suzhou, 215123
Jiangsu, P.R. China

Cell Line Authentication Service
STR Profile Report

Sales Order: 221202C

	Test Results for Submitted Sample		ExPASy Reference Database Profile	
**Loci**	**Query Profile:** Hep-G2/C3A		**Database Profile:** Hep-G2/C3A	
Amelogenin	X	Y	X	Y
D3S1358	15	16		
D5S818	11	13	11	13
D2S1338	19	20		
TPOX	8	9	8	9
CSF1PO	10	11	10	11
Penta D	9	13		
Y- Indel	2			
TH01	9		9	
vWA	17		17	
D7S820	10		10	
D21S11	29	31		
Penta E	15	20		
D10S1248	13			
D8S1179	15	16		
D1S1656	11	12		
D18S51	13	14		
D12S391	21	25		
D6S1043	13			
D19S433	15.2			
D16S539	12	13	12	13
D13S317	9	13	9	13
FGA	22	25		

The allele match algorithm compares the 8 core loci plus amelogenin only, even though alleles from all loci will be reported when available.

Note: Loci highlighted in grey (8 core STR loci plus Amelogenin) can be made public to verify cell identity. In order to protect the identity of the donor, ***please do not publish*** *the allele calls from all the STR loci tested.*

The sample match is based on the reference data available at the time of comparison.

Explanation of Test Results

Cell lines with ≥80% match are considered to be related; i.e., derived from a common ancestry. Cell lines with between a 55% to 80% match require further profiling for authentication of relatedness.

- [ ] The submitted sample profile is human, but not a match for any profile in the ExPASy STR database.
- [x] The submitted profile is an exact match for the following human cell line(s) in the ExPASy STR database (8 core loci plus Amelogenin): Hep-G2/C3A
- [ ] The submitted profile is similar to the following ExPASy human cell line(s):

e-Signature Technician: Digitally signed by Xuekun Chen
DN: cn=Xuekun Chen, o=Genetic Testing Biotechnology (Suzhou), ou=DNA Typing Section, email=order@jsdna.org, c=CN
Date: 2022.12.05 14:51:32 +08'00'

GENETIC TESTING BIOTECHNOLOGY — GUARANTEE — CELL LINE AUTHENTICATION

e-Signature Reviewer: Digitally signed by Xiankun Zhao
DN: cn=Xiankun Zhao, o=Genetic Testing Biotechnology (Suzhou), ou=Supervision Section, email=service@jsdna.org, c=CN
Date: 2022.12.05 14:53:38 +08'00'

More information

Addendum: Electropherogram for the customer's sample set 1 of 1

GENETIC TESTING BIOTECHNOLOGY

Cell Line Authentication Service
STR Profile Report

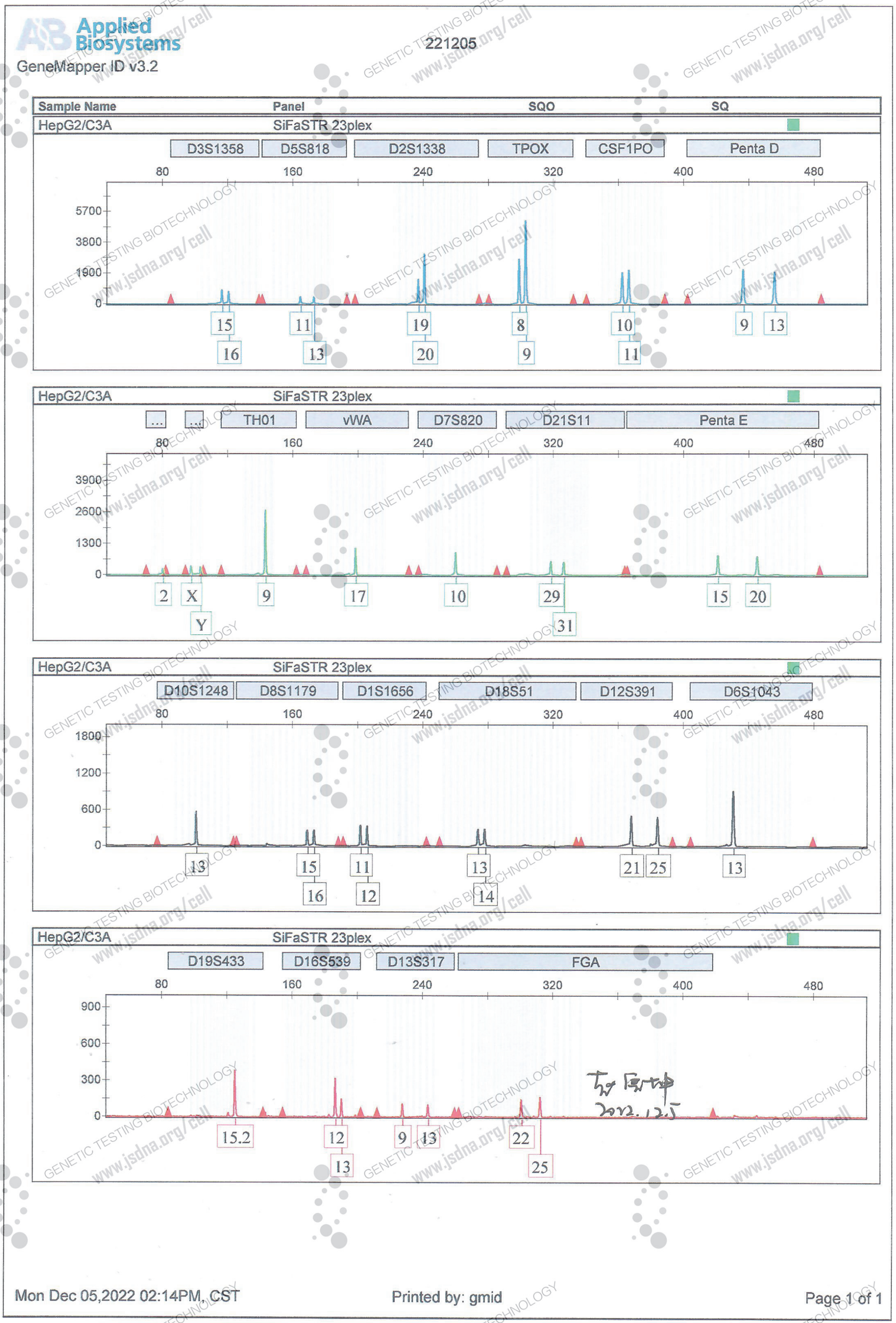

Cell Line Authentication Service
STR Profile Report

GENETIC TESTING BIOTECHNOLOGY

Sample Submitted By: Dr. Yingchao Wang
Mengchao Hepatobiliary Hospital of Fujian Medical University
Email Address: 15060092239@163.com
Sales Order: 221202D
Cell Line Designation: SNU-449
Date Sample Received: Dec 2th, 2022
Report Date: Dec 5th, 2022

Methodology: Twenty-one short tandem repeat (STR) loci, one Y-Indel locus plus the Amelogenin locus were amplified using the commercially available SiFaSTR™ 23 plex Kit. The cell line sample was processed using the ABI Prism® 3130 XL Genetic Analyzer. Data were analyzed using GeneMapper® ID v3.2 software (Applied Biosystems). Appropriate positive and negative controls were run and confirmed for each sample submitted.

Data Interpretation: Cell lines were authenticated using Short Tandem Repeat (STR) analysis as described in 2012 in ANSI Standard (ASN-0002) by the ATCC Standards Development Organization (SDO) and in Capes-Davis et al., Match criteria for human cell line authentication: Where do we draw the line? Int J Cancer. 2013;132(11):2510-9.

GTB™ performs STR Profiling following ISO 9001:2008 and ISO/IEC 17025:2005 quality standards.
There are no warranties with respect to the services or results supplied, express or implied, including, without limitation, any implied warranty of merchantability or fitness for a particular purpose. Genetic Testing Biotechnology (GTB) is not liable for any damages or injuries resulting from receipt and/or improper, inappropriate, negligent or other wrongful use of the test results supplied, and/or from misidentification, misrepresentation, or lack of accuracy of those results. Your exclusive remedy against GTB and those supplying materials used in the services for any losses or damage of any kind whatsoever, whether in contract, tort, or otherwise, shall be, at GTB's option, refund of the fee paid for such service or repeat of the service.

NOTE: According to the recommendations of *IJC* on cell line authentication, the report is valid for 3 years since the issue date.

Technical Questions?
GTB Technical Support
+86-512-67486171
service@jsdna.org
Section 505, Yixin BLD
SIP, Suzhou, 215123
Jiangsu, P.R. China

Ordering Questions?
order@jsdna.org
GTB Corporation
+86-512-62806339
Section 303, Yixin BLD
SIP, Suzhou, 215123
Jiangsu, P.R. China

GENETIC TESTING BIOTECHNOLOGY

Cell Line Authentication Service
STR Profile Report

Sales Order: 221202D

Loci	Test Results for Submitted Sample<br>Query Profile: SNU-449		ExPASy Reference Database Profile<br>Database Profile: SNU-449	
Amelogenin	X		X	
D3S1358	15	16		
D5S818	10		10	
D2S1338	19			
TPOX	11		11	
CSF1PO	11		11	
Penta D	10	11		
Y- Indel	-			
TH01	6	9	6	9
vWA	14	16	14	16
D7S820	8	13	8	13
D21S11	29	31		
Penta E	10	15		
D10S1248	12	15		
D8S1179	10	13		
D1S1656	15	17		
D18S51	14	16		
D12S391	17	18		
D6S1043	19			
D19S433	15.2	17.2		
D16S539	9		9	
D13S317	9		9	
FGA	22	24		

The allele match algorithm compares the 8 core loci plus amelogenin only, even though alleles from all loci will be reported when available.

Note: Loci highlighted in grey (8 core STR loci plus Amelogenin) can be made public to verify cell identity. In order to protect the identity of the donor, ***please do not publish*** *the allele calls from all the STR loci tested.*

The sample match is based on the reference data available at the time of comparison.

Explanation of Test Results

Cell lines with ≥80% match are considered to be related; i.e., derived from a common ancestry. Cell lines with between a 55% to 80% match require further profiling for authentication of relatedness.

☐ The submitted sample profile is human, but not a match for any profile in the ExPASy STR database.

☑ The submitted profile is an exact match for the following human cell line(s) in the ExPASy STR database (8 core loci plus Amelogenin): SNU-449

☐ The submitted profile is similar to the following ExPASy human cell line(s):

e-Signature Technician: Digitally signed by Xuekun Chen
DN: cn=Xuekun Chen, o=Genetic Testing Biotechnology (Suzhou), ou=DNA Typing Section, email=order@jsdna.org, c=CN
Date: 2022.12.05 14:52:49 +08'00'

GENETIC TESTING BIOTECHNOLOGY — GUARANTEE — CELL LINE AUTHENTICATION ★ ★ ★ ★ ★

e-Signature Reviewer: Digitally signed by Xiankun Zhao
DN: cn=Xiankun Zhao, o=Genetic Testing Biotechnology (Suzhou), ou=Supervision Section, email=service@jsdna.org, c=CN
Date: 2022.12.05 14:53:59 +08'00'

More information

Addendum: Electropherogram for the customer's sample set 1 of 1

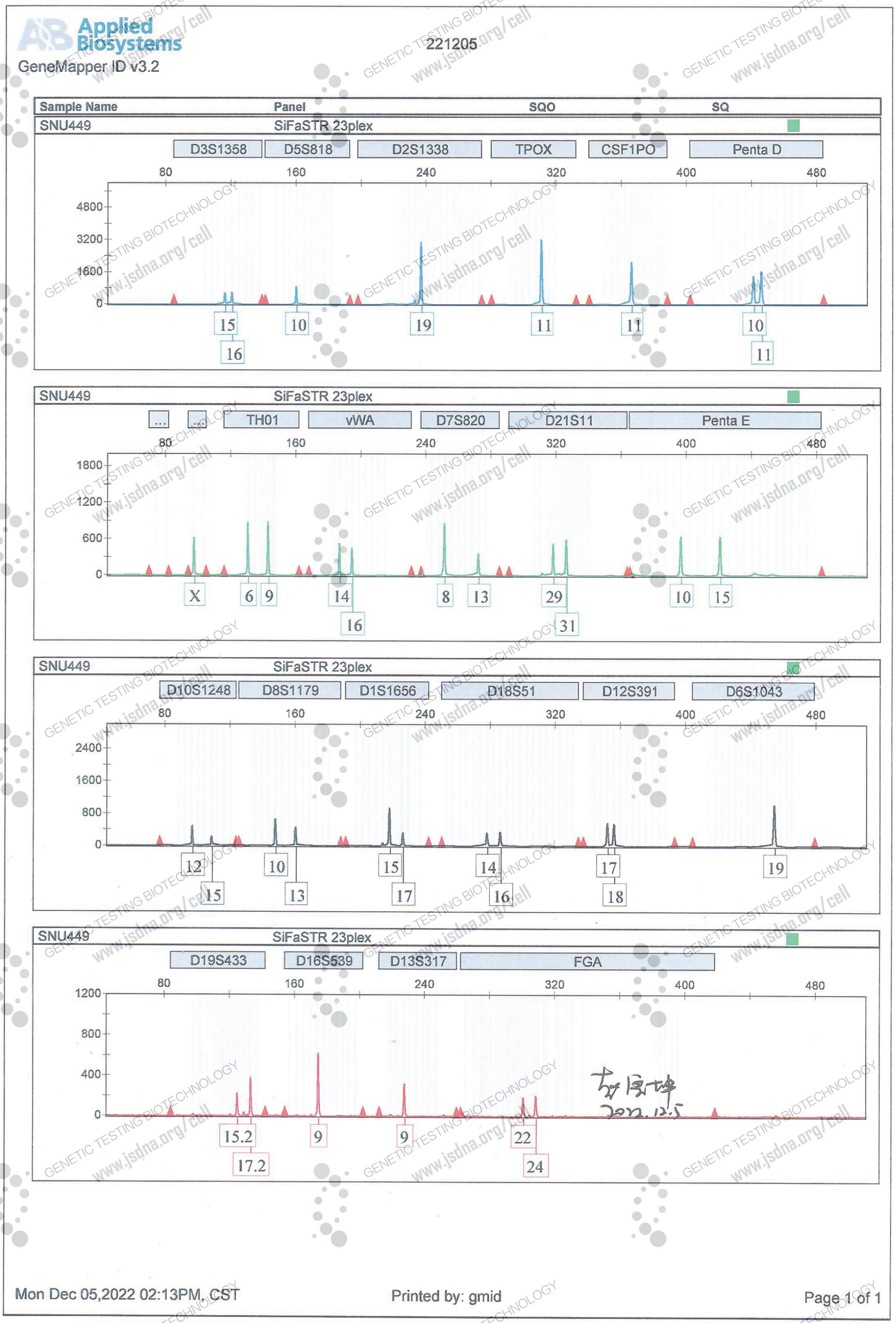
Applied Biosystems
GeneMapper ID v3.2
221205
Sample Name
Panel
SQO
SQ
SNU449
SiFaSTR 23plex
D3S1358
D5S818
D2S1338
TPOX
CSF1PO
Penta D
15
16
10
19
11
11
10
11
TH01
vWA
D7S820
D21S11
Penta E
X
6
9
14
16
8
13
29
31
10
15
D10S1248
D8S1179
D1S1656
D18S51
D12S391
D6S1043
12
15
10
13
15
17
14
16
17
18
19
D19S433
D16S539
D13S317
FGA
15.2
17.2
9
9
22
24
Mon Dec 05,2022 02:13PM, CST
Printed by: gmid
Page 1 of 1

GENEWIZ®

Solid science. Superior service.

Cell Line Authentication Report

GENEWIZ,Inc. Beijing

30 Science Park Road

Zhong-Guan-Cun Life Science Park

Changping District, 102206

Beijing, China

Tel: 400-8100-669

Fax: 010-59458058

Email: Genomics.China@genewiz.com.cn

www.genewiz.com.cn

GENEWIZ,Inc. Beijing
30 Life Science Park Road
Changping District, 102206
Beijing, China
Tel: 400-8100-669
Fax: 010-59458058
www.genewiz.com.cn

Cell Line Authentication Report
SSSS

Customer: CaiZhiXiong

Institution: Fuzhou city hospital of infectious diseases

Quotation Number: 80-33385805

Completion Date：04/06/2017

1. **Sample ID: SMMC-7721**
2. **Original Material: Cell pellets**
3. **Methods:**

1). Genomic DNA was extracted from the cell pellets provided by the customer.

2). Samples, together with positive and negative control were amplified using GenePrint 10 System (Promega).

3). Amplified products were processed using the ABI 3730xl Genetic Analyzer.

4). Data were analyzed using GeneMapper4.0 software and then compared with the ATCC, DSMZ or JCRB databases for reference matching.

4. **Results:**

1) **10 Loci STR Profile：**

Genetic Site	China Infrastruture of Cell Line Resources		Customer sample	
(Locus)	SMMC-7721		SMMC-7721	
Amelogenin	X		X	
CSF1PO	10		9	10
D13S317	13.3	15.1	12	13.3
D16S539	9	10	9	10
D5S818	11	12	11	12
D7S820	12		8	12
THO1	7		7	
TPOX	12		8	12
vWA	16	18	16	18
D21S11	27	28	27	28
Percent match between the sample and the database profile：84.85%				

GENEWIZ,Inc. Beijing
30 Life Science Park Road
Changping District, 102206
Beijing, China
Tel: 400-8100-669
Fax: 010-59458058
www.genewiz.com.cn

Summary:

1) Your cell line is considered "related" to the reference cell line in the ATCC STR database, as the STR profile yields matches that are ≥ 80% but less than 100%.

Notes：

1. P=100% x (2xM)/N; M=14, N=33 P=100% x (2x14)/33=84.85%

M: number of the matching peaks; N: number of all peaks

2. Based on the ANSI Standard, cell lines with ≥80% match are considered to be related; i.e.,derived from a common ancestry. Cell lines with between a 55% to 80% match require futher profiling for authentication of relatedness.

3. The short tandem repeat (STR) profile generated by GENEWIZ Inc. is indicative only of the sample sent to GENEWIZ Inc. at the time it was sent. This data and analysis are for research use only.

GENEWIZ,Inc. Beijing
30 Life Science Park Road
Changping District, 102206
Beijing, China
Tel: 400-8100-669
Fax: 010-59458058
www.genewiz.com.cn

2) Electrophoretogram

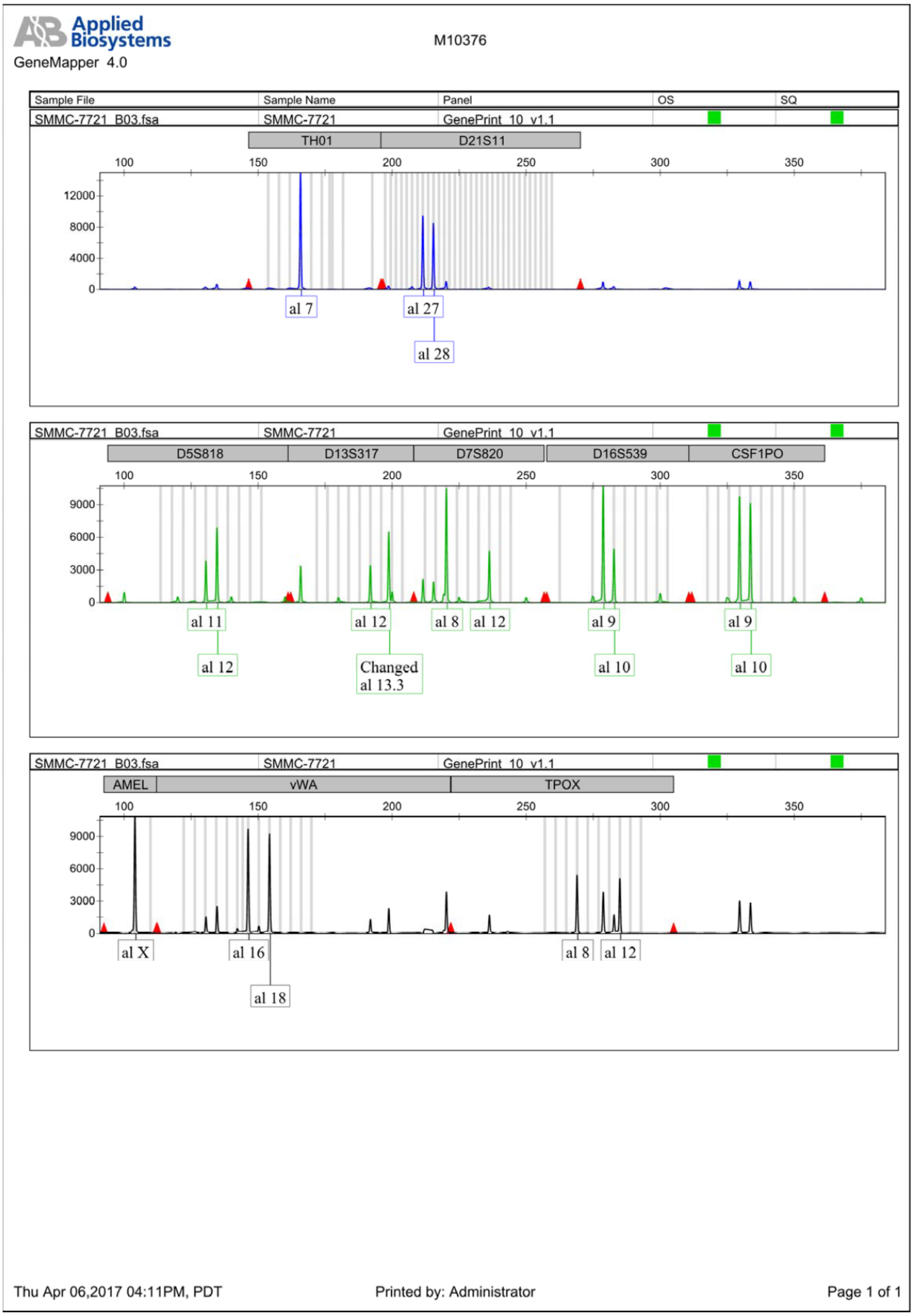

Note：Raw data in appendix
