# Supplementary material for: LncRNA TIALD contributes to hepatocellular carcinoma metastasis via inducing AURKA lysosomal degradation
Source: Cell Death Discov. 2023 Aug 26;9:316. doi: 10.1038/s41420-023-01620-w (PMC10541412; doi:10.1038/s41420-023-01620-w)

Original Western Blot

Figure 2E

Left

E-cadherin

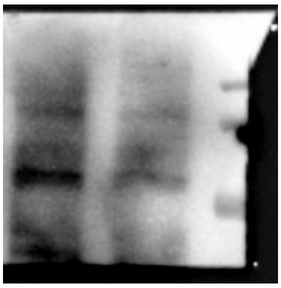

N-cadherin

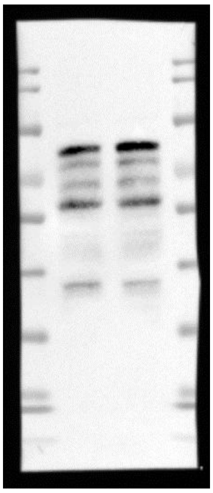

Snail

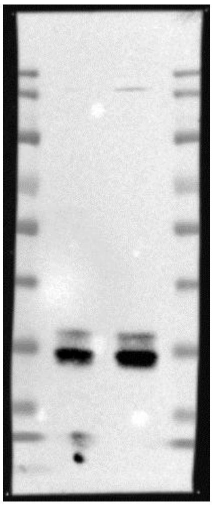

Slug

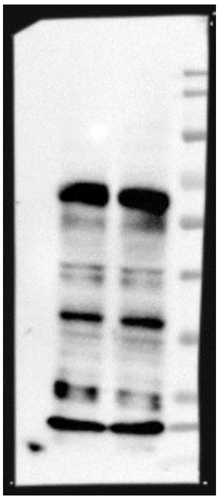

Actin

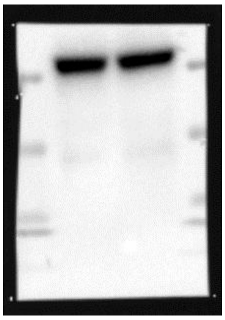

Right

E-cadherin

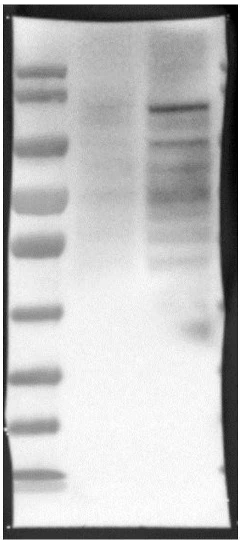

N-cadherin

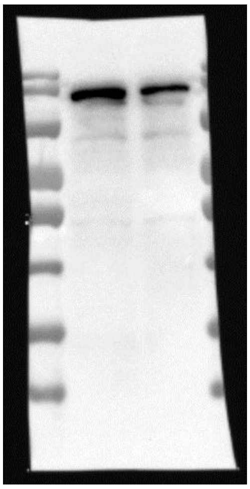

Snail

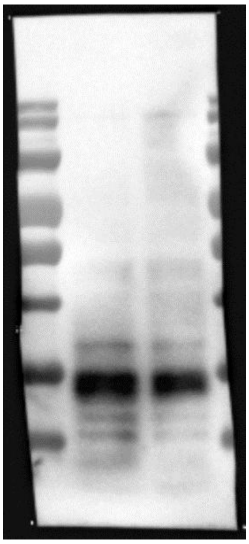

Slug

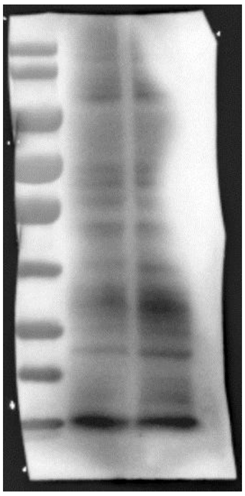

Actin

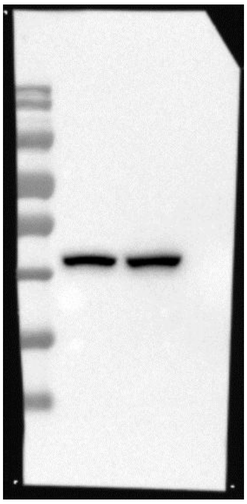

Figure 3B

SMMC-7721

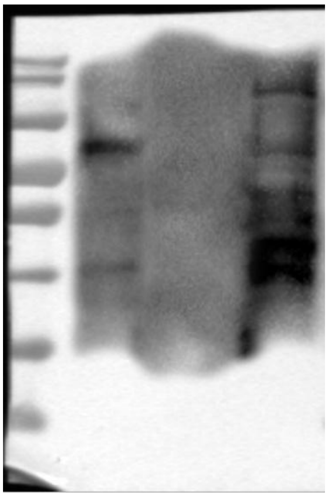

SNU449

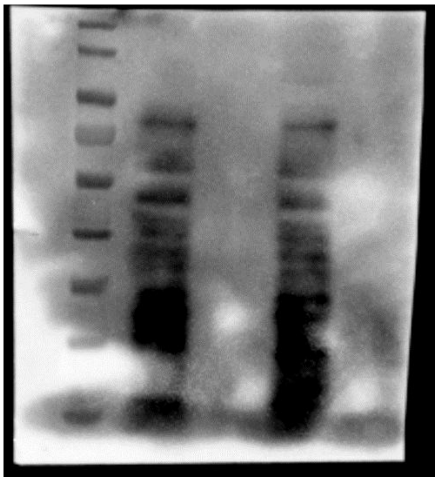

Figure 3D

SMMC-7721

METTL16

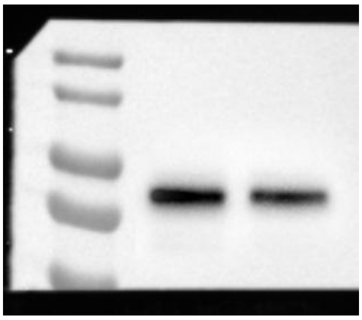

Actin

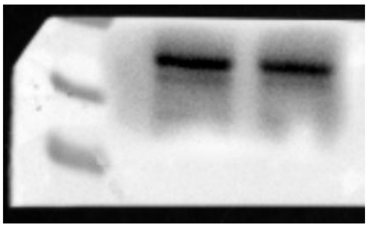

SNU449

METTL16

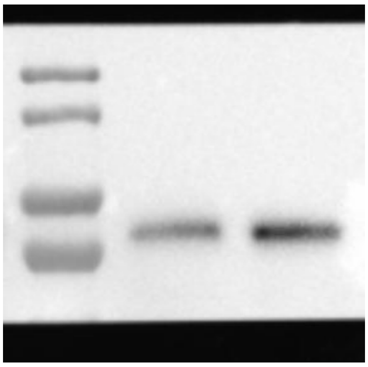

ACTIN

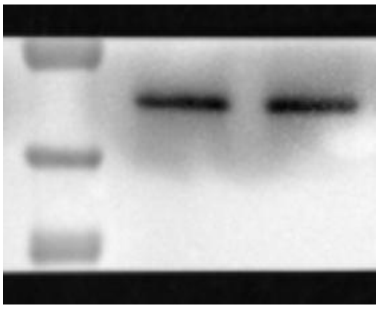

Figure 5A

SMMC-7721

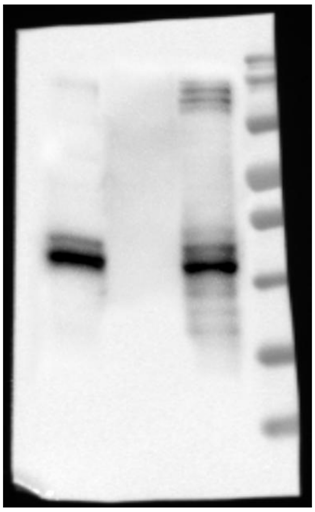

SNU449

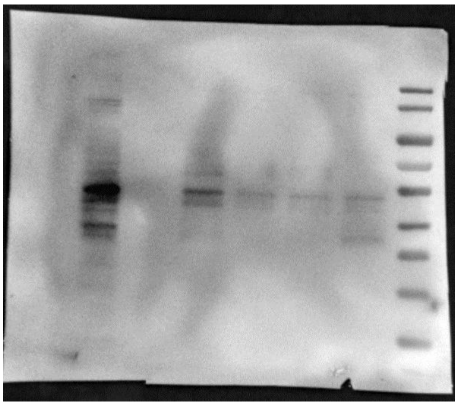

Figure 5C

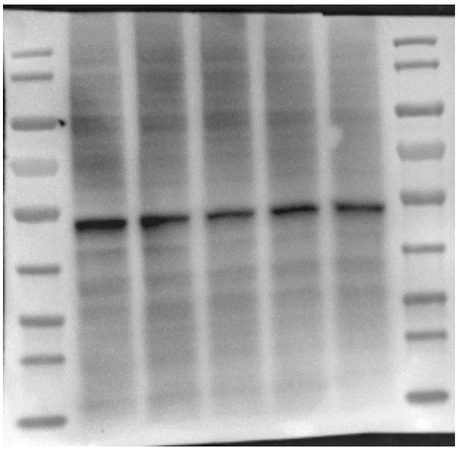

Figure 5E

SMMC-7721

AURKA

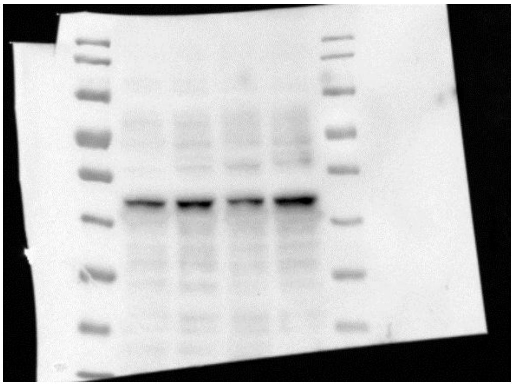

Actin

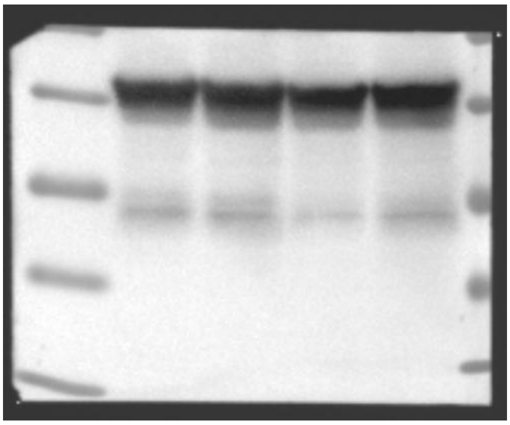

SNU449

AURKA

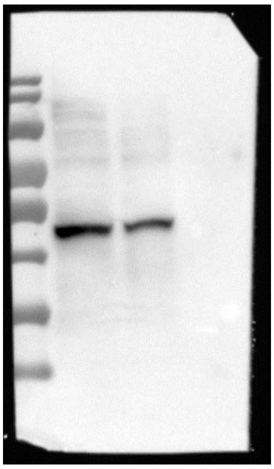

Actin

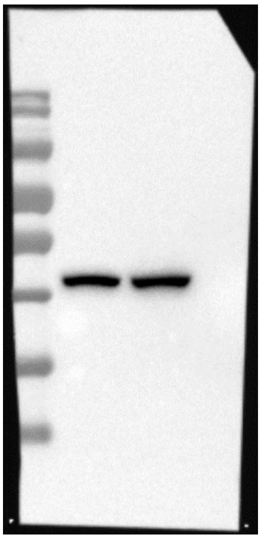

Figure 5G

shNC

AURKA

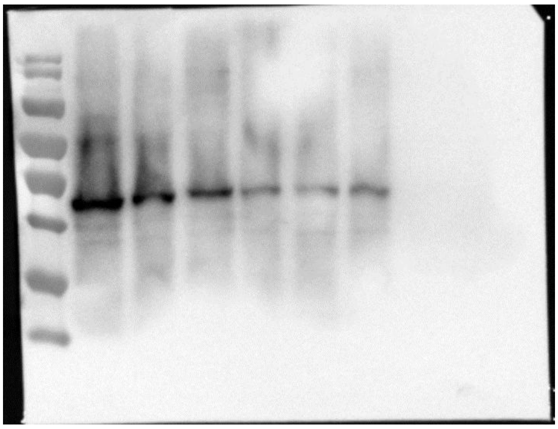

Actin

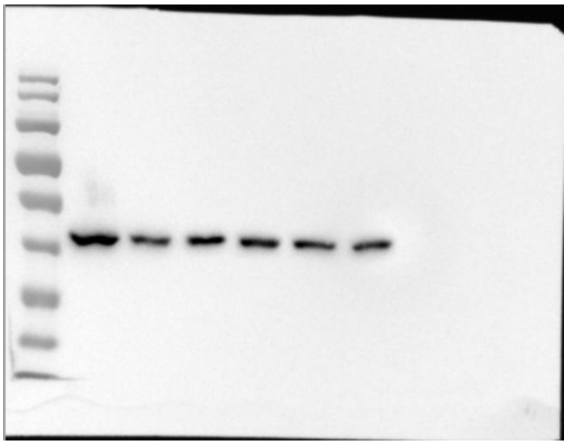

shTIALD

AURKA

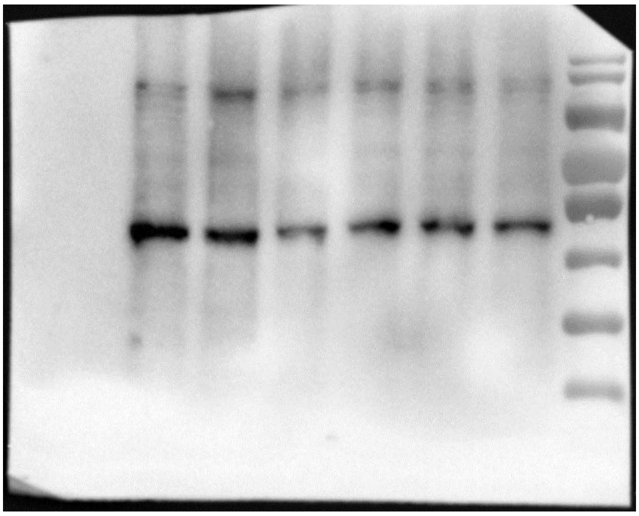

Actin

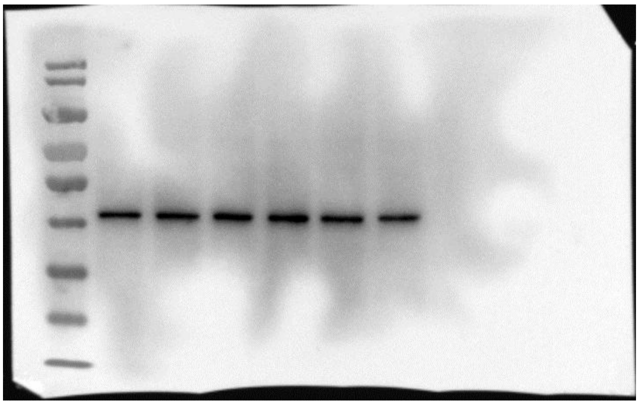

Ctrl

AURKA

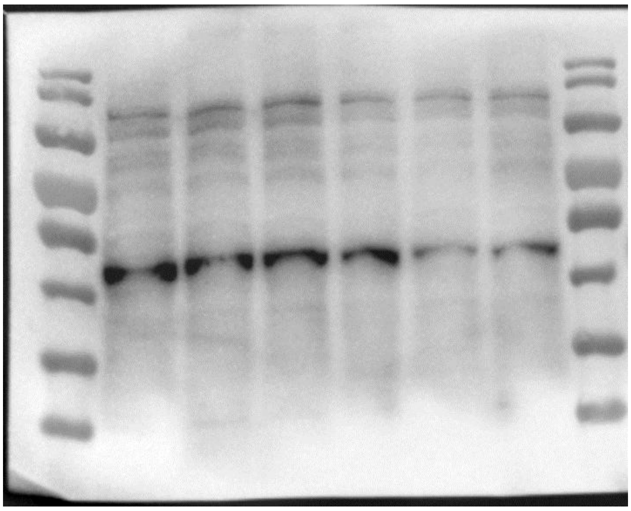

Actin

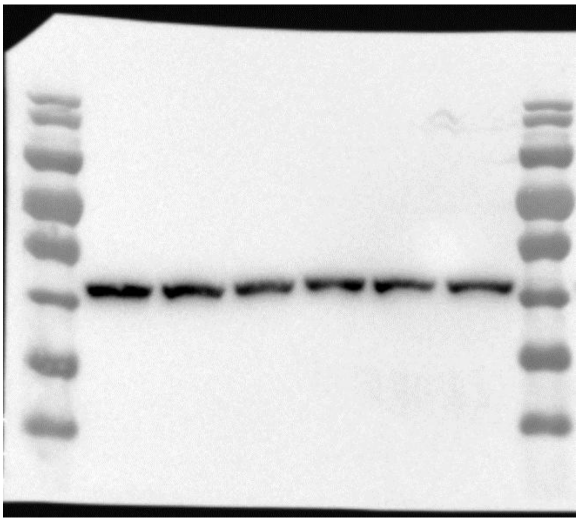

TIALD

AURKA

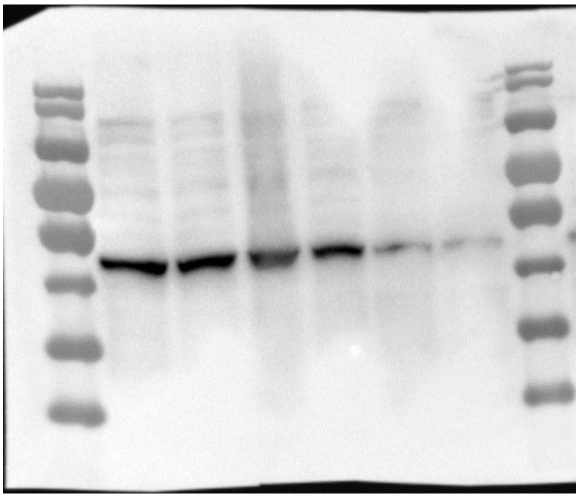

Actin

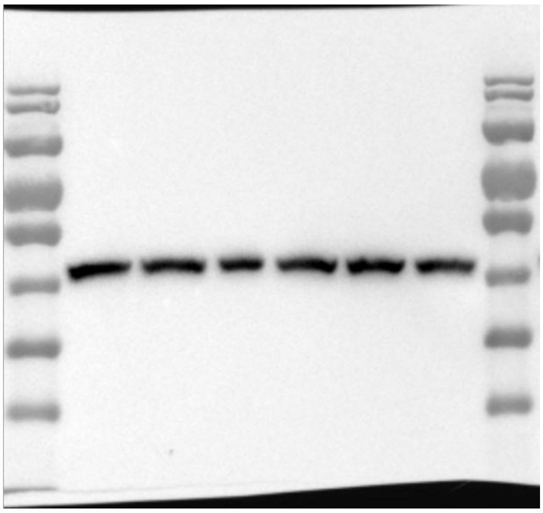

Figure 5H

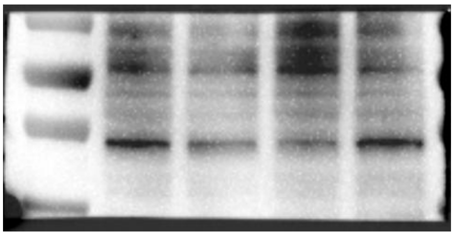

Figure 6C

Left

AURKA

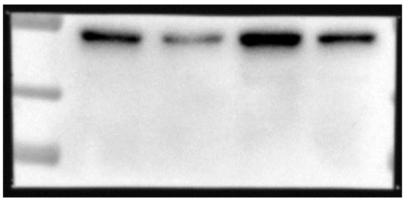

E-Cadherin

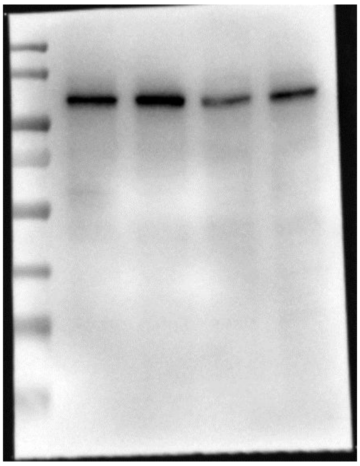

N-Cadherin

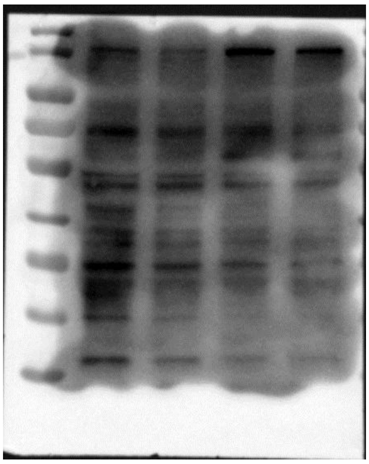

Snail

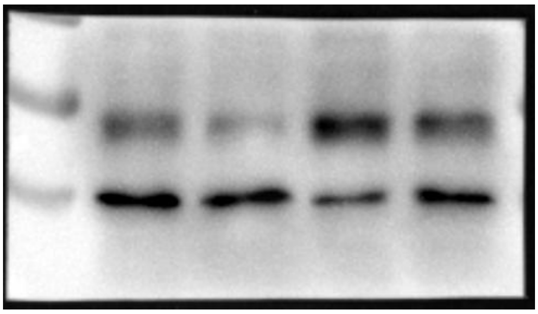

Slug

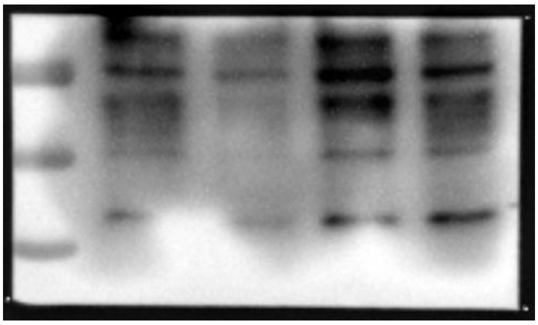

Actin

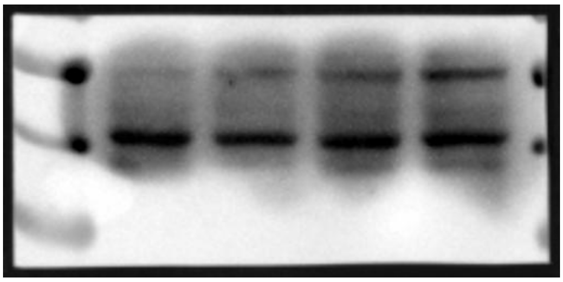

Right

AURKA

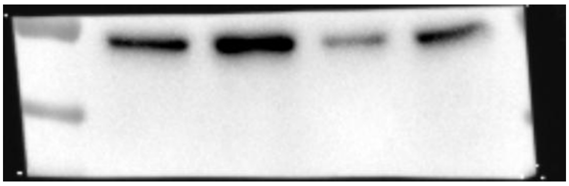

E-Cadherin

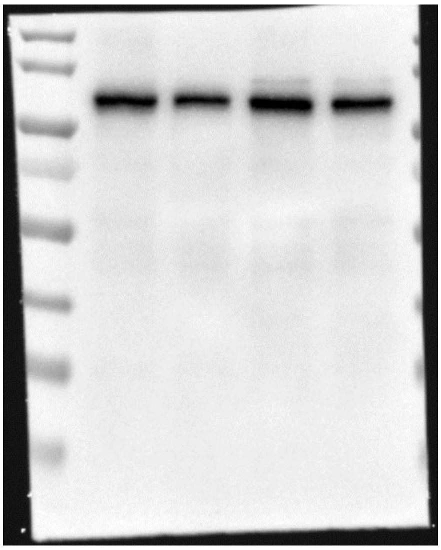

N-Cadherin

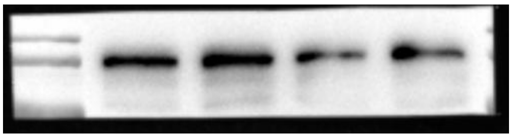

Snail

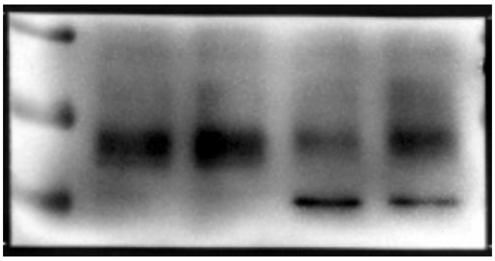

Slug

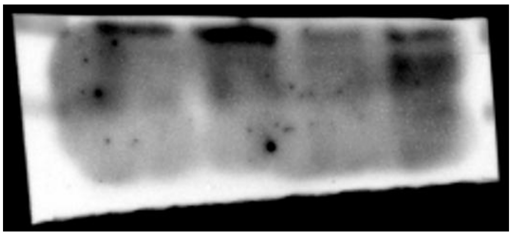

Supplement: Supplementary file 3 — Original Data File [file 41420_2023_1620_MOESM3_ESM.pdf]
